# Gaze and Movement Assessment (GaMA): Inter-site validation of a visuomotor upper limb functional protocol

**DOI:** 10.1371/journal.pone.0219333

**Published:** 2019-12-30

**Authors:** Heather E. Williams, Craig S. Chapman, Patrick M. Pilarski, Albert H. Vette, Jacqueline S. Hebert

**Affiliations:** 1 Department of Mechanical Engineering, Faculty of Engineering, University of Alberta, Edmonton, Alberta, Canada; 2 Faculty of Kinesiology, Sport, and Recreation, University of Alberta, Edmonton, Alberta, Canada; 3 Department of Medicine, Faculty of Medicine and Dentistry, University of Alberta, Edmonton, Alberta, Canada; 4 Department of Biomedical Engineering, Faculty of Medicine and Dentistry, University of Alberta, Edmonton, Alberta, Canada; 5 Glenrose Rehabilitation Hospital, Alberta Health Services, Edmonton, Alberta, Canada; University of Exeter, UNITED KINGDOM

## Abstract

**Background:**

Successful hand-object interactions require precise hand-eye coordination with continual movement adjustments. Quantitative measurement of this visuomotor behaviour could provide valuable insight into upper limb impairments. The Gaze and Movement Assessment (GaMA) was developed to provide protocols for simultaneous motion capture and eye tracking during the administration of two functional tasks, along with data analysis methods to generate standard measures of visuomotor behaviour. The objective of this study was to investigate the reproducibility of the GaMA protocol across two independent groups of non-disabled participants, with different raters using different motion capture and eye tracking technology.

**Methods:**

Twenty non-disabled adults performed the Pasta Box Task and the Cup Transfer Task. Upper body and eye movements were recorded using motion capture and eye tracking, respectively. Measures of hand movement, angular joint kinematics, and eye gaze were compared to those from a different sample of twenty non-disabled adults who had previously performed the same protocol with different technology, rater and site.

**Results:**

Participants took longer to perform the tasks versus those from the earlier study, although the relative time of each movement phase was similar. Measures that were dissimilar between the groups included hand distances travelled, hand trajectories, number of movement units, eye latencies, and peak angular velocities. Similarities included all hand velocity and grip aperture measures, eye fixations, and most peak joint angle and range of motion measures.

**Discussion:**

The reproducibility of GaMA was confirmed by this study, despite a few differences introduced by learning effects, task demonstration variation, and limitations of the kinematic model. GaMA accurately quantifies the typical behaviours of a non-disabled population, producing precise quantitative measures of hand function, trunk and angular joint kinematics, and associated visuomotor behaviour. This work advances the consideration for use of GaMA in populations with upper limb sensorimotor impairment.

## Introduction

Various sensorimotor impairments including stroke [1], amputation [2], and spinal cord injury [3] lead to deficits in upper limb performance, which can hamper activities of daily living that require precise hand-object interactions [4]. Functional assessments are used to gauge the impact of upper limb impairment and to monitor rehabilitative progress thereafter [5,6]. However, such assessments often do not yield precise and comprehensive measures of joint and trunk movements, along with hand function measures such as grip aperture [7,8]. Furthermore, they do not tend to measure the corresponding hand-eye interaction, which is recognized as an important behaviour during grasp control [9,10]. Quantitative measurement of visuomotor behaviour collected during the execution of functional tasks can enhance the understanding of these movement features. Measurement technologies commonly used for this purpose include eye tracking and motion capture. Existing assessments reliant on such specialized equipment, however, can be criticized as not being generalizable to authentic activities of daily function, as they tend to focus on simple functions of reaching and grasping [11–19]. Furthermore, technology-based assessments risk becoming obsolete as newer technologies emerge, hindering the opportunity for robust comparisons over time.

A Gaze and Movement Assessment (GaMA) protocol was developed, based on the foundation of visuomotor research that both acknowledges and demonstrates a means of overcoming these limitations [7–9]. GaMA uses motion capture and eye tracking to quantify the movement quality and visual attention exhibited by participants as they interact with and move objects in an environment. GaMA includes: (1) a procedure for the administration of two standardized functional tasks that incorporate common dextrous hand demands of daily living–the ‘Pasta Box Task’ and ‘Cup Transfer Task’ [7]; (2) a methodology to obtain synchronized motion capture and eye tracking data during functional task execution [7–9]; and (3) analysis software, which requires a standardized data set of synchronized movement and eye data coordinates as input, and outputs measures of hand movement, angular joint kinematics, and eye gaze [7–9].

The standardized ‘Pasta Box Task’ and ‘Cup Transfer Task’ used in GaMA were designed based on common task requirements of functional clinical assessments, and were shown to have repeatable hand trajectory and hand kinematics among able-bodied participants [7]. Motion capture marker clusters are used to reduce the implementation burden, particularly as such clusters were validated as being equivalent to individually placed anatomical markers [20]. Consistent normative joint movement kinematics for GaMA’s functional tasks have been demonstrated, with test-retest reliability in a normative population [8]. Movement and eye data synchronization and analysis have established that participants’ visual attention to future actions was similar between participants and across tasks [9], thereby reinforcing the theoretical framework of visual allocation during goal-directed actions [21].

GaMA’s analysis software requires a specific input data set that can be obtained by various data collection hardware and software solutions. This renders GaMA amenable to technological evolution, such as markerless motion capture and mobile eye trackers. Furthermore, GaMA’s output measures of hand movement, angular joint kinematics, and eye gaze (which can be precisely reported for individual movement phases) remain relevant and equipment-independent for future comparative purposes, both within and across research sites. The ability to compare results across sites would be extremely valuable, as it could facilitate larger subgroup comparisons when smaller populations of individuals with upper limb impairments are studied, such as upper limb prosthesis users.

In order to validate a new protocol such as GaMA, it is essential to determine reproducibility. Reproducibility of a test or method is defined as the closeness of the agreement between independent results obtained by following the same procedures, but under different experimental conditions [22]. Due to the inherent variability found in clinical populations, reproducibility of a test to assess movement behaviour is typically first studied in a non-disabled population. While intra-rater test-retest reliability of GaMA has been demonstrated for hand movement and angular joint kinematic measures for non-disabled individuals [7,8], it has yet to be determined whether these and other measures obtainable by GaMA are reproducible across raters and sites. Furthermore, it is often assumed that the non-disabled population will behave similarly (or identically) across test sites; yet, it is known that deviations from protocols can result in data set disparity amongst the population [23]. If a standardized protocol can be shown to yield measures that are similar across sites, the data sets could be combined for a richer understanding (or more saturated data set) of non-disabled movement behaviour.

The objective of this study, therefore, was to conduct an inter-site validation of GaMA by assessing the reproducibility of the visuomotor measures in non-disabled individuals presented by Valevicius et al. and Lavoie et al. [7–9]. More specifically, this study sought to determine whether the same hand movement, angular joint kinematic, and eye gaze measures could be obtained by testing a second independent group of non-disabled participants, at a different site equipped with different motion capture and eye tracking technology, and administered by a different rater. Establishing the reproducibility of GaMA in the non-disabled population advances its consideration as an outcome assessment protocol for populations with sensorimotor impairments of the upper limb.

## Methods

For comparative purposes, the research conducted by Valevicius et al. [7,8] and Lavoie et al. [9] is referred to in this paper as ‘the original study’, and the data set analyzed by these studies is referred to as ‘the original data set’. The new research presented in this article is referred to as ‘the repeated study’ and its data as ‘the repeated data set’. Unless otherwise specified, the same procedures were followed in both studies. Further details about such procedures can be found in the GaMA protocol supplementary materials (S1 Text). Ethical approval for these procedures was obtained by the University of Alberta Health Research Ethics Board (Pro00054011), the Department of the Navy Human Research Protection Program, and the SSC-Pacific Human Research Protection Office.

### Participants

A total of 22 non-disabled adults were recruited to participate in the repeated study. Data from two participants were removed due to problems arising from software issues. The characteristics of the 20 participants from the original study [7–9] and the 20 participants in the repeated study are detailed in Table 1. In both studies, two participants performed the tasks without corrected vision, since they had to remove their glasses to don the eye tracker. These participants, however, reported that their vision was sufficient to allow them to confidently perform the task.

**Table 1:** Participant characteristics in the original and repeated studies.

Research Participant Characteristics	Original Study	Repeated Study
Male participants	11	13
Female participants	9	7
Self-reported right-handed participants	18	19
Participants with normal or corrected to normal vision	18	18
Participant age (years–mean ± standard deviation)	25.8 ± 7.2	24.4 ± 7.3
Participant height (cm–mean ± standard deviation)	173.8 ± 8.3	171.0 ± 7.7

### Equipment

**Fig 1:**
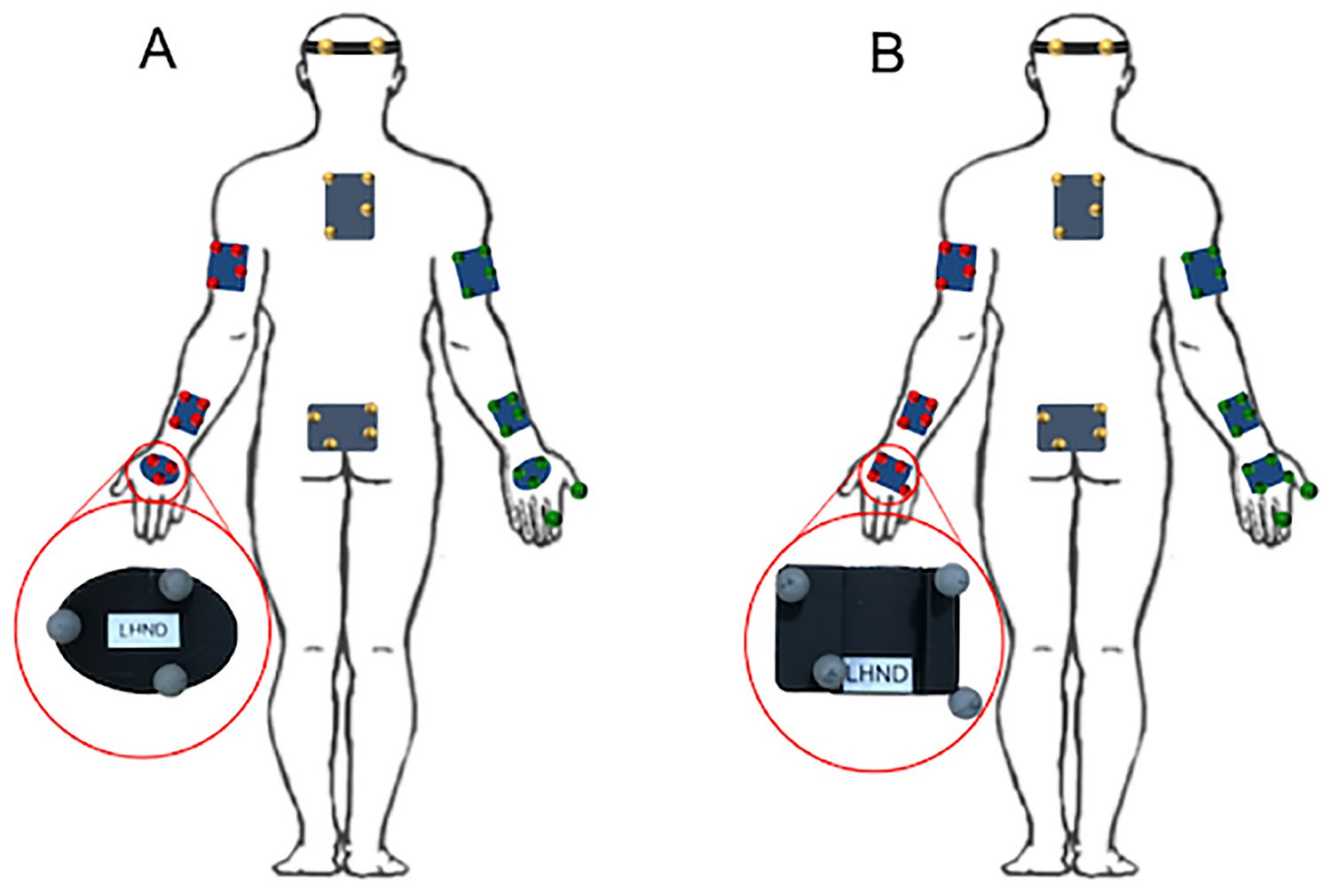
Retroreflective marker placement. Marker placement for participants in the original study (A) and repeated study (B), showing differences in the hand marker plate designs.

Motion capture and eye tracking hardware and software specifications for the original study and the repeated study are indicated in Table 2. The equipment was set up in the repeated study as specified in the original study [7–9]. Rigid plates and a headband (each holding four retroreflective markers) were attached to the participant in accordance with Boser et al.’s *Clusters Only* kinematic model [20]. To improve rigid body motion tracking in the repeated study, the hand plates were redesigned, as shown in Fig 1. For both studies, markers were attached to the index finger (middle phalange) and thumb (distal phalange) [7]; a head-mounted eye tracker was placed on the participant and positioned in accordance with the manufacturer’s instructions; and a motion capture calibration pose was collected for each participant, as outlined by Boser et al. [20].

**Table 2:** Specifications of the motion capture and eye tracking systems used in the original and repeated studies.

Specifications	Original Study	Repeated Study
Motion capture camera	Vicon Bonita 10 (Vicon Motion Systems Ltd, Oxford, UK)	OptiTrack Flex 13 (Natural Point, OR, USA)
Number of cameras	12	8
Camera sampling frequency	120 Hz	120 Hz
Head-mounted binocular eye tracker	Dikablis Professional 2 (Ergoneers GmbH, Manching, Germany)	Pupil (Pupil Labs GmbH, Berlin, Germany)
Eye camera sampling frequency	60 Hz	120 Hz

### Data collection

In both studies, the two functional tasks introduced by Valevicius et al. (the Pasta Box Task and Cup Transfer Task) [7] were administered. The Pasta Box Task involves the movement of a pasta box between a side table to shelves in front of the participant. The Cup Transfer Task involves the movement of two deformable cups filled with beads over a barrier on a tabletop in front of the participant. Additional details about these tasks can be found in Supplement S1. Each participant completed 20 error-free trials of the two tasks (if an error occurred, data from this erroneous trial were discarded and an additional trial was completed to replace it), while simultaneous motion and eye tracking data were collected. Prior to this, each participant was given verbal instructions, a demonstration, and at least one familiarization trial of each functional task. Task order was randomized for each participant. At least two gaze calibrations (outlined by Lavoie et al. [9]) were collected before participants executed their initial trial of each task, and one after they completed their final trial of the last task; given that there were two functional tasks, a minimum of 5 calibrations were performed per participant.

The original data collection protocol differed from the repeated study in one notable way. In the original study, every participant performed a total of 60 trials of each task, 20 of which were under each of the following conditions: (1) only motion capture data were collected, (2) only eye tracking data were collected, and (3) both motion capture and eye tracking data were collected. As the repeated study consisted solely of collecting data during simultaneous motion capture and eye tracking, it was only compared to that of the original data set captured under condition (3) ‘both’. In the original study, the order of conditions for each participant was block randomized to one of 4 block orders, with motion (1) and both (3) conditions always sequential. As a consequence of the partial randomization order, three quarters of the original-study participants were afforded at least 20 extra trials executing each functional task prior to testing under the ‘both’ condition.

### Experimental data analysis

Data analysis in the repeated study was undertaken as outlined by Valevicius et al. and Lavoie et al. [7–9], with details provided in Supplement S1. The data analysis was dependent on accurate and combined visuomotor data. This required that the motion capture marker trajectory data and pupil position data first be cleaned, filtered, and synchronized. Motion capture data cleaning was necessary to fill any gaps in the data. Pattern-based interpolation was used to fill all gaps originating from markers that were part of clusters, whereas linear or cubic interpolation was used for individual marker data. Pupil position data were cleaned using linear interpolation of gaps smaller than 100 ms. Next, the visuomotor data were filtered to reduce any noise that was introduced during data collection. Filtering was accomplished using second-order, low-pass Butterworth filters, with a cut-off frequency of 6 Hz for the motion capture data [7,8] and 10 Hz for eye tracking [9]. Finally, the motion capture and eye tracking data were synchronized.

**Fig 2:**
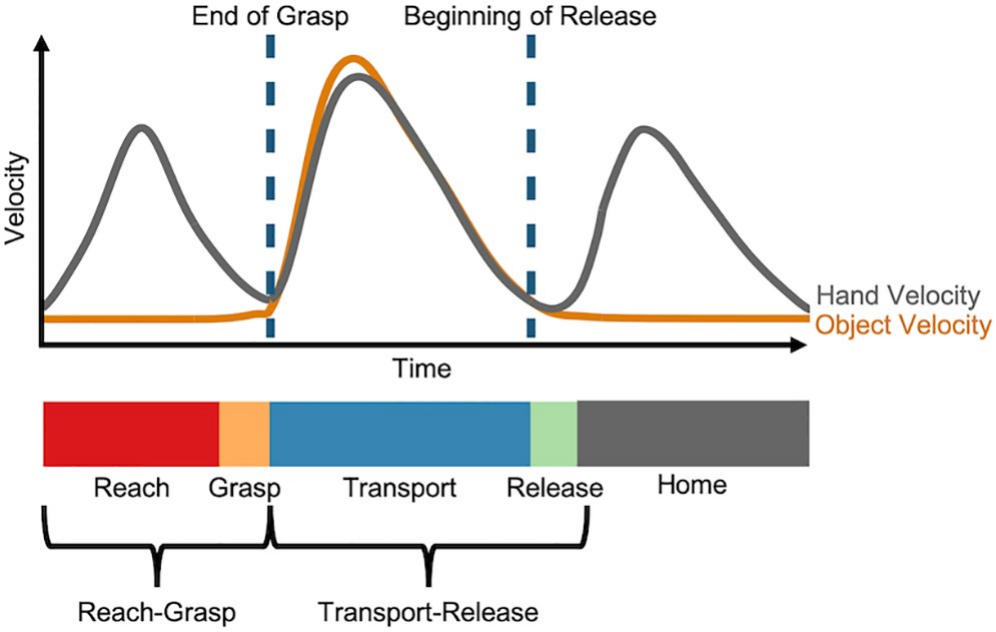
Phase transitions, phases, and movement segments within one movement. To illustrate how segmentation was carried out, representative average hand and object velocity profiles are displayed in grey and orange lines, respectively. Reach, Grasp, Transport and Release phases are presented along the bar as red, orange, blue and green, respectively. Home (grey bar) refers to the standardized location to which the hand returns at the completion of the movement.

For each functional task, the repeated data set were divided into distinct *movements* based on hand velocity, the velocity of the task object(s), and grip aperture values, as per Valevicius et al. [7]. The data from each movement were further segmented into the *phases* of ‘Reach’, ‘Grasp’, ‘Transport’, ‘Release’, and ‘Home’; the Home phase was not used for data analysis. Due to the short duration of the Grasp and Release phases, combined *movement segments* of ‘Reach-Grasp’ and ‘Transport-Release’ were used in hand movement analysis [7]. Eye latency measures were calculated at instances of *phase transition*, both at the end of a Grasp phase and at the beginning of a Release phase (referred to as ‘Pick-up’ and ‘Drop-off’ by Lavoie et al. [9]). An illustration of how one distinct movement was separated into the abovementioned subsets (phases, movement segments, and phase transitions) can be found in Fig 2.

### GaMA measures

Duration (phase and trial), hand movement, angular joint kinematic, and eye gaze measures were calculated for the original and repeated studies, as outlined by Valevicius et al. [7,8] and Lavoie et al. [9], and are listed in Table 3. Lavoie et al.’s ‘fixations to future’ measure was not considered in this study as these fixations were shown to be unlikely to occur in non-disabled participants for both tasks [9]. In addition to the measures listed in Table 3, the relative duration of each phase was calculated as the percent of time spent in that phase, relative to the given Reach-Grasp-Transport-Release sequence.

**Table 3:** Comparative measures, including duration, hand movement, angular joint kinematic, and eye gaze measures, and the subsets of each movement for which they were calculated.

Type of Measure	Measures	Movement Subsets
Duration (from Lavoie et al. [9])	Phase duration	Reach, Grasp, Transport, Release
Hand movement (from Valevicius et al. [7])	Hand distance travelled Hand trajectory variability Peak hand velocity Percent-to-peak hand velocity Number of movement units	Reach-Grasp, Transport-Release
	Peak grip aperture Percent-to-peak grip aperture Percent-to-peak hand deceleration	Reach-Grasp
Angular joint kinematics (from Valevicius et al. [8])	Peak angle, range of motion, and peak angular velocity for the following degrees of freedom: Trunk flexion/extension Trunk lateral bending Trunk axial rotation Shoulder flexion/extension Shoulder abduction/adduction Shoulder internal/external rotation Elbow flexion/extension Forearm pronation/supination Wrist flexion/extension Wrist ulnar/radial deviation	Movement only
Eye gaze (from Lavoie et al. [9])	Percent fixation to Current Number of fixations to Current	Reach, Grasp, Transport, Release
	Percent fixation to Hand in Flight Number of fixations to Hand in Flight	Reach, Transport
	Eye Arrival Latency Eye Leaving Latency	End of Grasp, Beginning of Release

In the repeated study, the calculation of hand movement measures was altered due to the creation of a virtual rectangular prism, which approximated the participant’s hand position at each point in time. Using the centre of this prism, hand position and velocity were subsequently calculated. For comparative purposes, the original study’s hand movement results were recalculated via this methodology, rather than the original calculation of Valevicius et al. that used the average position of the three hand plate markers [7]).

### Statistical analysis

The aim of the statistical analysis was to detect significant differences between the original and repeated data sets, and to determine whether such differences were more pronounced for particular movements and/or movement subsets (phase, movement segment, or phase transition). To investigate differences between the two groups of participants, a series of repeated-measures analyses of variance (RMANOVAs) and pairwise comparisons were conducted for each measure and task. RMANOVA group effects or interactions involving group were followed up with either an additional RMANOVA or pairwise comparisons between groups if the Greenhouse-Geisser corrected *p* value was less than 0.05. Pairwise comparisons were considered to be significant if the Bonferroni corrected *p* value was less than 0.05. Detailed statistical analysis methods can be found in supplementary materials (S2 Text).

## Results

### Duration

For both the Pasta Box Task (or ‘Pasta’) and the Cup Transfer Task (or ‘Cups’), the repeated-study participants took significantly more time to complete the tasks than the original-study participants (Pasta: 11.8 ± 3.4 seconds versus 8.8 ± 1.2 seconds, p < 0.01; Cups: 13.9 ± 2.5 seconds versus 10.5 ± 1.3 seconds, p < 0.0001). The repeated-study participants had longer phase durations than the original-study participants, with all Grasp and Transport phases and the Movement 2 Release phase significantly prolonged in Pasta, and all phases significantly prolonged in Cups (S1 Table). The two participant groups, however, displayed similar relative phase durations throughout both tasks, with no significant differences.

### Hand movement

**Fig 3:**
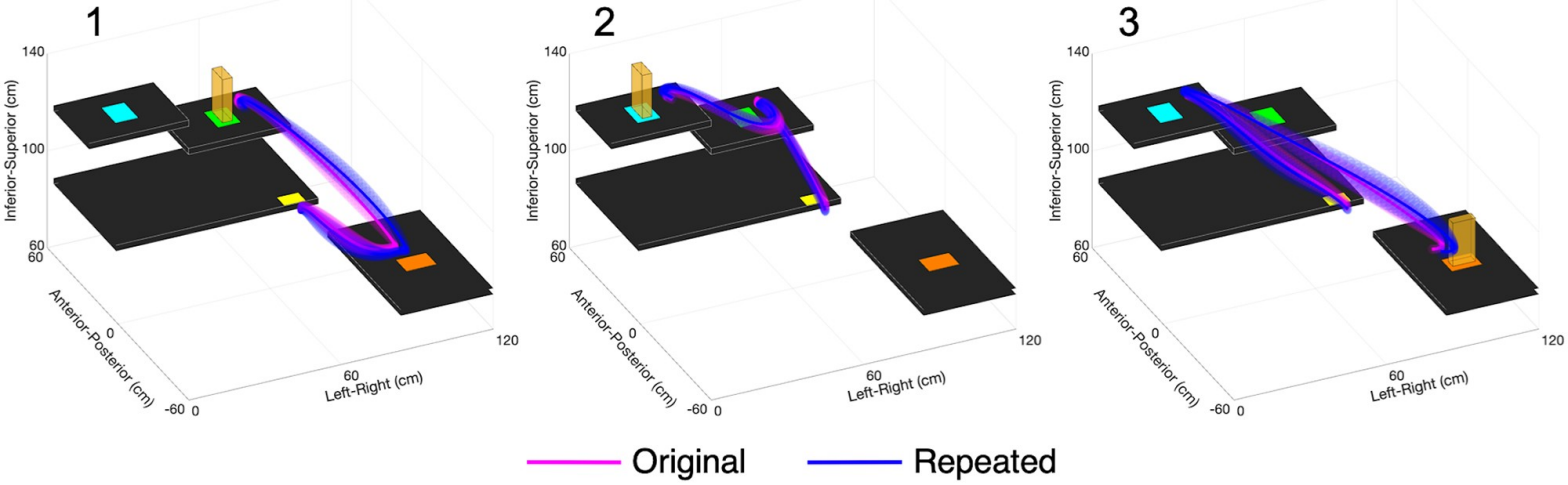
Pasta Box Task hand trajectories. Trajectories are displayed for participants in the original (pink) and repeated (blue) studies for Movements 1, 2, and 3. The solid lines represent participant group averages, and the three-dimensional shading represents the standard deviation of participant group means.

**Fig 4:**
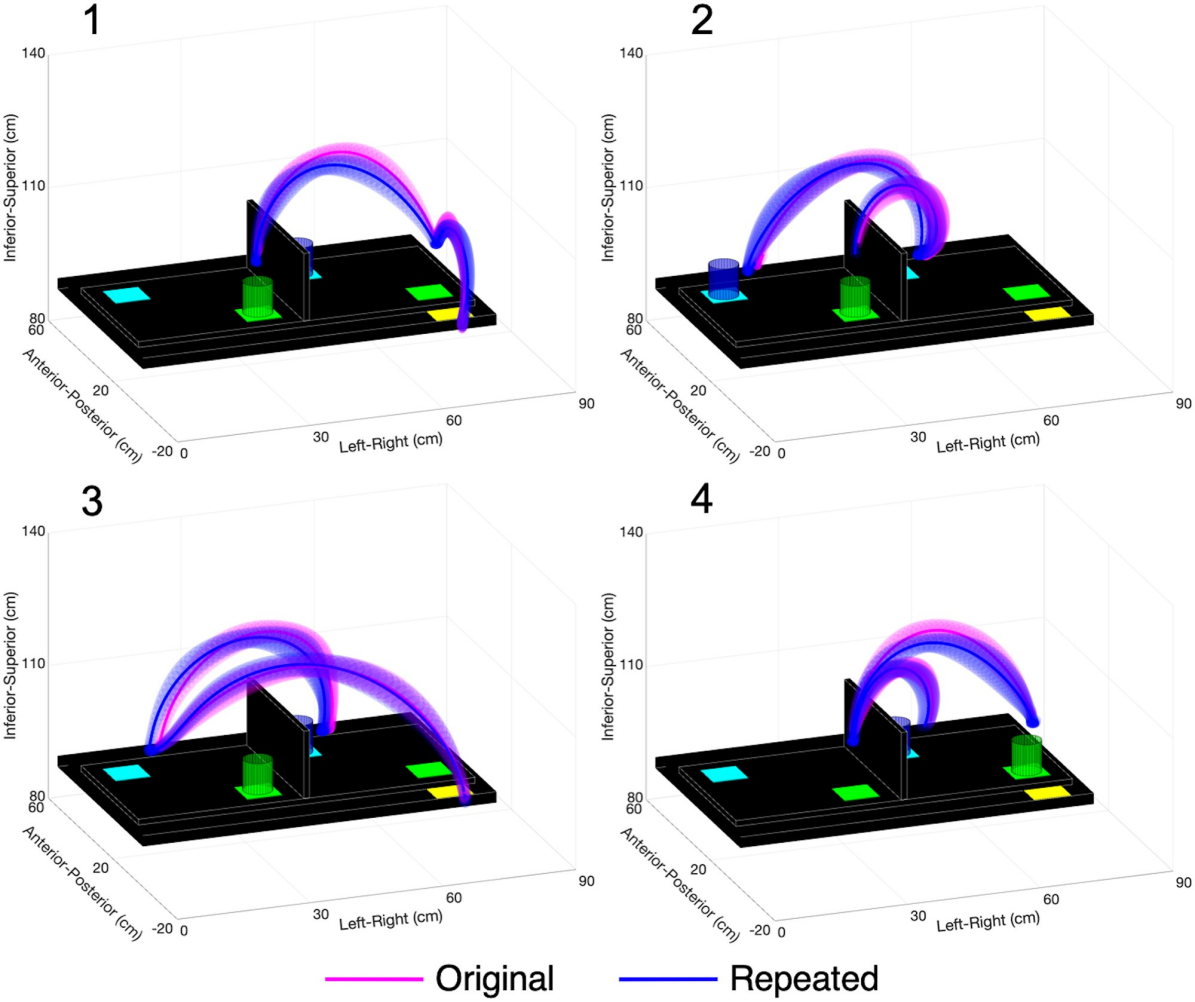
Cup Transfer Task hand trajectories. Trajectories are displayed for participants in the original (pink) and repeated (blue) studies for Movements 1, 2, 3, and 4. The solid lines represent participant group averages, and the three-dimensional shading represents the standard deviation of participant group means.

The repeated-study participants had greater hand distances travelled than the original-study participants, with significant increases in Movement 1 & 3 segments of Pasta (S2 Table) and in all Cups movement segments, except for Movement 1 & 4 Transport-Releases (S3 Table). However, Fig 3 (Pasta) and Fig 4 (Cups) show that the average hand trajectories chosen by both participant groups were similar. The repeated-study participants also had larger hand trajectory variability than the original-study participants, with significant increases in all Pasta movement segments except for Movement 3 Transport-Release (S2 Table) and all Cups movement segments (S3 Table). The repeated-study participants had a greater number of movement units (i.e., more hand velocity peaks) than the original-study participants, with significant increases in all movement segments of Pasta and for Movement 1 & 4 Reach-Grasp and Movement 1 to 3 Transport-Release segments of Cups.

**Fig 5:**
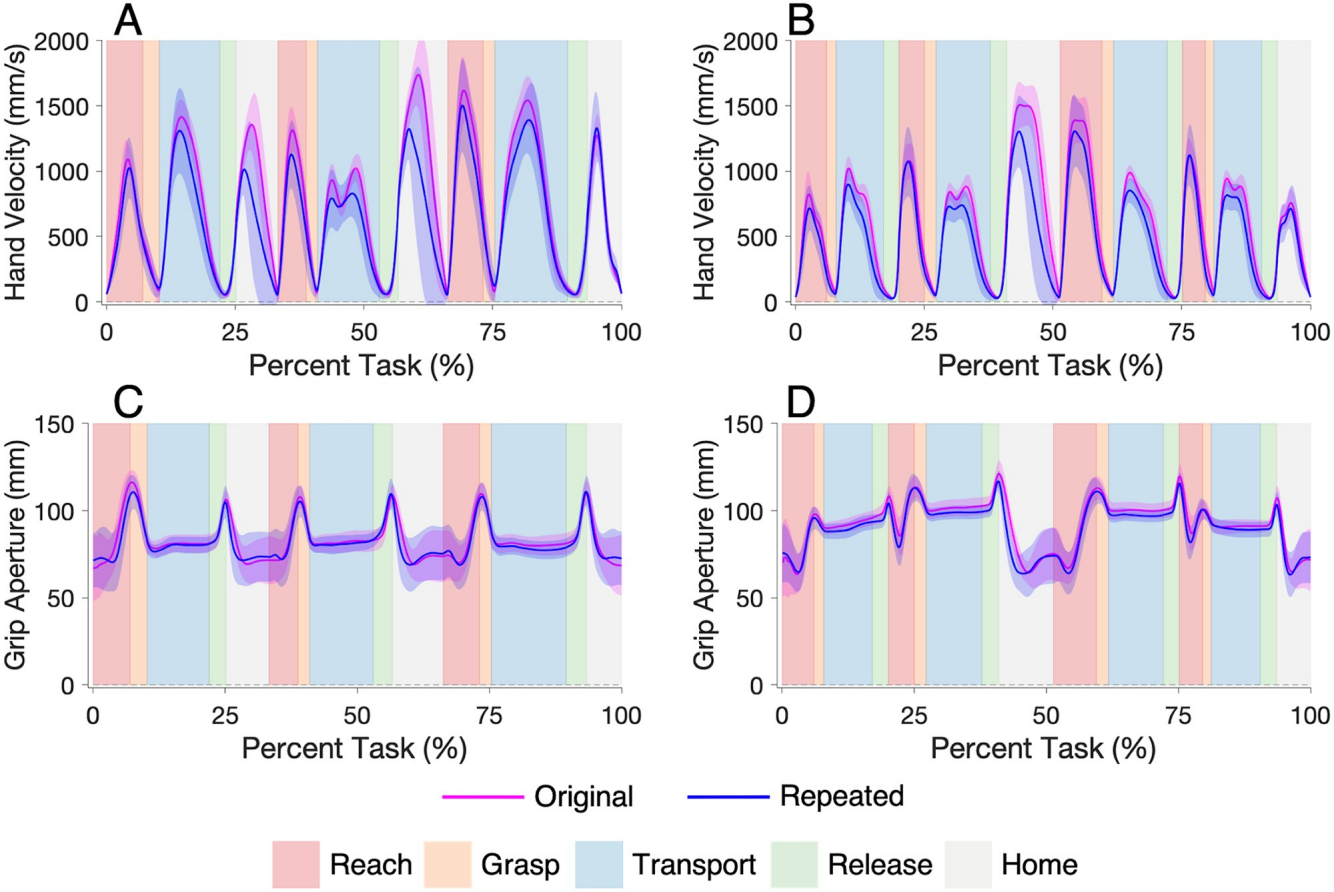
Hand velocity profiles for the Pasta Box Task (A) and the Cup Transfer Task (B); and grip aperture profiles for the Pasta Box Task (C) and the Cup Transfer Task (D). Original-study data are presented in pink, and repeated-study data in blue. The solid lines represent participant group averages, and the shading represents one standard deviation of the participant group means. Each task is segmented into Reach (red), Grasp (orange), Transport (blue), Release (green), and Home (grey) phases for each movement.

Participants in the original and repeated studies had similar hand velocity profiles for both tasks, as shown in Fig 5A and 5B. Although the peaks in the repeated study appeared smaller, these differences were non-significant throughout both tasks (S2 and S3 Tables). Significant percent-to-peak hand velocity differences were identified for the Movement 1 Reach-Grasp segment of Pasta and the Movement 2 & 3 Reach-Grasp segments of Cups, but the differences between the mean values of the two participant groups were less than one standard deviation of the original study results. Participants in the original and repeated studies showed similar percent-to-peak hand deceleration values, with no significant differences in Pasta and a significantly difference only for the Movement 4 Reach-Grasp segment of Cups. However, the difference between the mean values of the two participant groups in this movement segment was less than one original study standard deviation.

Participants in the original and repeated studies had similar grip aperture profiles for both tasks, as shown in Fig 5C and 5D, with no significant differences in peak grip aperture identified for either task. Also, no significant differences in percent-to-peak grip aperture were identified in Pasta, and a significant difference was only identified in the Movement 4 Reach-Grasp segment of Cups.

### Angular joint kinematics

**Fig 6:**
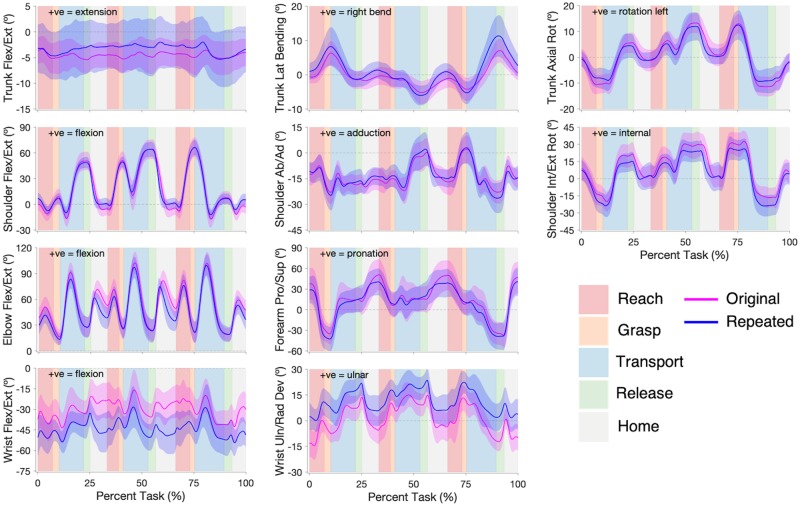
Pasta Box Task angular joint trajectories. Original (pink) and repeated (blue) studies angular joint trajectories for trunk flexion/extension, lateral bending, and axial rotation; shoulder flexion/extension, abduction/ adduction, and internal/external rotation; elbow flexion/extension and forearm pronation/supination; and wrist flexion/extension and ulnar/radial deviation. The solid lines represent participant group averages, and the shading represents one standard deviation of the participant group means. The task is segmented into Reach (red), Grasp (orange), Transport (blue), Release (green), and Home (grey) phases for each movement.

**Fig 7:**
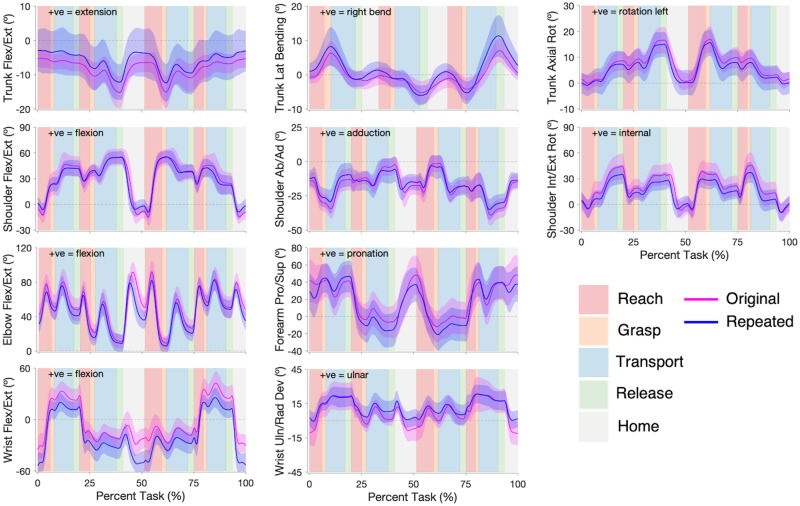
Cup Transfer Task angular joint trajectories. Original (pink) and repeated (blue) studies angular joint trajectories for trunk flexion/extension, lateral bending, and axial rotation; shoulder flexion/extension, abduction/ adduction, and internal/external rotation; elbow flexion/extension and forearm pronation/supination; and wrist flexion/extension and ulnar/radial deviation. The solid lines represent participant group averages, and the shading represents one standard deviation of the participant group means. The task is segmented into Reach (red), Grasp (orange), Transport (blue), Release (green), and Home (grey) phases for each movement.

Angular kinematic trajectories illustrating the average joint trajectories of participants are shown in Fig 6 (Pasta) and Fig 7 (Cups). Similar angular kinematic profiles existed between the original- and repeated-study participants, with only a few differences; participants in the repeated study had an increased standard deviation for trunk flexion/extension (both tasks), and an offset was present between the wrist flexion/extension angles (both tasks) and between the wrist ulnar/radial deviations angles (Pasta only) of the two participant groups. Angular kinematic measures are presented in Table 4 (Pasta) and Table 5 (Cups). The original- and repeated-study participants generally had similar peak joint angles in both tasks. Significant peak angle differences were found in wrist flexion/extension for Movements 1 and 2 of Pasta and all movements of Cups, and in wrist ulnar/radial deviation for all movements of Pasta.

**Table 4:** Pasta Box Task angular joint kinematic values (presented as means ± between-participant standard deviations), with significant results of the RMANOVAs and pairwise comparisons.

		Peak Angle (degrees)			Range of Motion (degrees)			Peak Angular Velocity (degrees/s)		
	M	*p*	Original	Repeated	*p*	Original	Repeated	*p*	Original	Repeated
**T** **F** **E**	1	ns	-2.1 ± 2.4	-0.9 ± 4.7	ns	4.9 ± 1.6	6.0 ± 2.2	ns	18.8 ± 5.4	23.7 ± 7.8
	2	ns	-2.7 ± 2.6	0.2 ± 5.0	*	3.6 ± 1.0	5.8 ± 2.5	*	14.9 ± 5.4	22.9 ± 8.4
	3	ns	-2.1 ± 2.5	0.2 ± 5.0	ns	4.9 ± 1.4	7.3 ± 4.2	*	18.2 ± 5.0	28.4 ± 13.6
**T** **L** **B**	1	ns	6.5 ± 3.5	8.6 ± 5.4	ns	8.7 ± 2.8	10.2 ± 4.2	ns	21.7 ± 5.5	19.9 ± 8.0
	2	ns	0.2 ± 2.5	1.7 ± 2.4	*	5.6 ± 2.0	8.2 ± 2.6	ns	12.8 ± 3.6	15.0 ± 3.7
	3	ns	7.2 ± 3.5	11.6 ± 6.0	*	11.8 ± 2.8	17.3 ± 6.6	ns	21.3 ± 3.9	24.2 ± 6.6
**T** **A** **R**	1	ns	6.0 ± 3.9	5.0 ± 4.6	ns	17.8 ± 2.4	14.9 ± 4.9	ns	42.6 ± 6.6	37.0 ± 11.1
	2	ns	13.7 ± 3.9	12.4 ± 5.5	ns	15.1 ± 3.0	13.8 ± 4.6	ns	33.4 ± 8.3	36.8 ± 13.6
	3	ns	13.3 ± 3.8	12.9 ± 5.8	ns	25.5 ± 3.0	24.1 ± 8.2	ns	58.6 ± 10.8	52.3 ± 17.1
**S** **F** **E**	1	ns	51.3 ± 10.6	49.3 ± 6.5	ns	69.3 ± 7.6	61.4 ± 8.5	*	192.3 ± 39.4	143.8 ± 44.1
	2	ns	64.9 ± 11.4	64.7 ± 8.7	ns	72.1 ± 9.7	67.1 ± 9.9	*	200.8 ± 40.9	154.1 ± 45.0
	3	ns	66.8 ± 11.2	67.0 ± 8.8	ns	86.0 ± 9.9	81.7 ± 10.1	ns	233.0 ± 40.4	192.8 ± 54.2
**S** **A** **A**	1	ns	-5.8 ± 5.1	-6.1 ± 6.8	ns	19.3 ± 6.5	20.1 ± 5.2	ns	76.6 ± 23.6	65.6 ± 27.0
	2	ns	1.4 ± 7.2	3.1 ± 9.4	ns	25.6 ± 8.8	25.6 ± 8.0	ns	81.5 ± 30.7	69.7 ± 21.3
	3	ns	3.5 ± 6.9	4.0 ± 8.6	ns	28.9 ± 9.1	32.0 ± 10.5	ns	101.7 ± 27.6	90.0 ± 24.3
**S** **I** **E** **R**	1	ns	22.8 ± 10.0	16.4 ± 8.4	ns	44.0 ± 7.9	41.5 ± 9.2	*	151.1 ± 32.3	112.5 ± 40.8
	2	ns	32.6 ± 10.4	27.3 ± 9.6	ns	32.6 ± 6.7	27.8 ± 6.9	*	123.3 ± 23.1	89.4 ± 34.4
	3	ns	34.9 ± 9.6	29.7 ± 9.7	ns	54.2 ± 6.8	55.7 ± 10.2	ns	180.4 ± 33.8	148.9 ± 44.4
**E** **F** **E**	1	ns	92.1 ± 11.9	85.4 ± 11.5	ns	76.4 ± 10.6	73.1 ± 10.2	*	274.2 ± 53.8	218.5 ± 62.1
	2	ns	103.6 ± 12.8	98.6 ± 12.7	ns	81.2 ± 9.6	78.8 ± 9.6	ns	268.1 ± 47.5	226.1 ± 51.4
	3	ns	103.8 ± 13.2	102.3 ± 12.2	ns	88.4 ± 11.6	87.4 ± 11.3	ns	270.3 ± 48.6	226.8 ± 55.2
**F** **P** **S**	1	ns	40.1 ± 22.5	33.8 ± 20.2	ns	77.0 ± 15.9	78.9 ± 19.0	*	308.6 ± 70.4	244.7 ± 72.5
	2	ns	51.3 ± 22.3	44.7 ± 20.2	ns	51.4 ± 18.2	47.1 ± 12.4	ns	176.4 ± 57.6	149.2 ± 51.7
	3	ns	51.4 ± 21.7	42.7 ± 19.9	ns	90.9 ± 17.3	85.3 ± 16.4	ns	181.8 ± 47.9	169.5 ± 62.2
**W** **F** **E**	1	*	-18.6 ± 12.4	-29.1 ± 8.7	ns	28.6 ± 6.1	31.0 ± 8.4	*	136.8 ± 30.4	109.3 ± 27.2
	2	*	-11.8 ± 13.8	-23.5 ± 12.2	ns	25.5 ± 8.9	32.0 ± 10.3	ns	122.3 ± 36.4	119.6 ± 37.0
	3	ns	-12.6 ± 11.4	-22.5 ± 15.3	ns	32.3 ± 8.0	36.4 ± 14.7	ns	123.9 ± 38.6	123.0 ± 42.6
**W** **U** **R** **D**	1	*	14.6 ± 7.8	*23.1 ± 7.1	ns	30.9 ± 5.6	25.7 ± 7.4	*	108.9 ± 39.3	77.7 ± 30.1
	2	*	18.8 ± 7.8	*26.4 ± 6.8	ns	24.7 ± 7.3	22.4 ± 7.6	*	95.6 ± 23.0	69.1 ± 24.1
	3	*	16.3 ± 7.3	*24.6 ± 7.0	ns	29.7 ± 4.7	26.4 ± 5.8	*	117.5 ± 28.0	88.8 ± 30.8

Angular kinematic values include peak angle (degrees), range of motion (degrees), and peak angular velocity (degrees/s) of each movement (M) for trunk flexion/extension (TFE), lateral bending (TLB), and axial rotation (TAR); shoulder flexion/extension (SFE), abduction/adduction (SAA), and internal/external rotation (SIER); elbow flexion/extension (EFE) and forearm pronation/supination (FPS); and wrist flexion/extension (WFE) and ulnar/radial deviation (WURD). For the results of the pairwise comparisons (in column *p*),

* indicates a *p* value less than 0.05,

** indicates a *p* value less than 0.005, and

ns indicates a *p* value that is not significant.

Highlighted table cells also indicate significant differences (red = higher and blue = lower repeated study value).

**Table 5:** Cup Transfer Task angular joint kinematic values (presented as means ± between-participant standard deviations), with significant results of the RMANOVAs and pairwise comparisons.

		Peak Angle (degrees)			Range of Motion (degrees)			Peak Angular Velocity (degrees/s)		
	M	*p*	Original	Repeated	*p*	Original	Repeated	*p*	Original	Repeated
**T** **F** **E**	1	ns	-4.4 ± 2.5	-1.2 ± 6.2	*	3.0 ± 1.5	4.7 ± 1.9	**	10.7 ± 3.4	16.0 ± 4.7
	2	ns	-6.3 ± 2.5	-2.9 ± 5.7	ns	9.1 ± 3.3	10.2 ± 2.5	ns	23.1 ± 6.8	25.2 ± 6.4
	3	ns	-5.6 ± 2.8	-2.1 ± 6.1	ns	9.6 ± 3.1	11.2 ± 3.2	ns	27.2 ± 7.8	31.8 ± 12.2
	4	ns	-5.7 ± 2.7	-3.0 ± 6.5	ns	4.7 ± 2.5	6.0 ± 1.5	ns	13.0 ± 4.1	16.6 ± 5.3
**T** **L** **B**	1	ns	-0.4 ± 1.8	1.9 ± 3.9	**	4.8 ± 1.9	7.7 ± 2.3	ns	9.9 ± 3.6	12.0 ± 3.4
	2	ns	0.3 ± 4.1	1.9 ± 4.6	ns	7.2 ± 2.4	9.5 ± 3.6	ns	16.5 ± 6.0	19.0 ± 8.1
	3	ns	-0.6 ± 3.2	2.2 ± 4.1	**	6.2 ± 1.9	9.4 ± 3.3	ns	15.1 ± 3.7	20.9 ± 10.3
	4	ns	-1.1 ± 3.0	0.8 ± 4.4	*	4.0 ± 1.4	5.7 ± 2.0	ns	10.8 ± 3.4	12.9 ± 5.3
**T** **A** **R**	1	ns	8.9 ± 3.7	7.7 ± 5.1	ns	9.3 ± 2.5	9.2 ± 2.7	ns	20.6 ± 4.0	21.5 ± 5.2
	2	ns	17.1 ± 5.0	15.7 ± 4.7	ns	10.7 ± 2.8	11.3 ± 3.4	ns	28.1 ± 7.4	30.1 ± 9.3
	3	ns	17.2 ± 5.0	16.4 ± 5.0	ns	16.7 ± 4.2	16.9 ± 4.7	ns	39.1 ± 9.8	44.2 ± 15.9
	4	ns	10.3 ± 3.9	8.6 ± 4.8	ns	7.9 ± 2.4	7.2 ± 2.3	ns	22.6 ± 7.3	22.2 ± 6.7
**S** **F** **E**	1	ns	49.2 ± 14.6	43.9 ± 9.1	*	62.7 ± 13.5	50.8 ± 11.4	*	142.1 ± 42.8	104.5 ± 31.7
	2	ns	56.8 ± 10.4	55.7 ± 7.3	ns	30.9 ± 6.2	29.6 ± 4.9	ns	103.9 ± 29.7	77.9 ± 31.7
	3	ns	57.5 ± 11.0	56.4 ± 7.5	ns	73.6 ± 10.4	66.6 ± 8.9	*	228.2 ± 58.8	174.3 ± 61.6
	4	ns	49.5 ± 14.8	43.7 ± 10.7	ns	29.6 ± 9.0	26.4 ± 8.6	ns	104.7 ± 25.0	95.2 ± 34.5
**S** **A** **A**	1	ns	-8.1 ± 4.7	-6.3 ± 6.2	ns	27.5 ± 7.1	23.7 ± 5.4	ns	80.4 ± 23.4	65.8 ± 19.7
	2	ns	-1.4 ± 5.9	-3.4 ± 8.2	ns	18.7 ± 5.6	16.2 ± 4.2	*	63.7 ± 17.1	49.3 ± 14.3
	3	ns	0.1 ± 5.5	-1.1 ± 7.7	ns	28.7 ± 8.6	23.9 ± 6.3	ns	98.1 ± 34.9	79.3 ± 33.0
	4	ns	-13.9 ± 6.9	-14.4 ± 7.1	ns	26.1 ± 6.0	21.7 ± 5.8	*	74.9 ± 19.4	59.3 ± 16.5
**S** **I** **E** **R**	1	ns	44.9 ± 14.9	35.5 ± 10.9	ns	51.5 ± 13.9	41.2 ± 10.6	*	116.0 ± 57.8	74.1 ± 23.8
	2	ns	43.6 ± 13.8	34.8 ± 10.2	ns	33.1 ± 7.5	28.2 ± 6.5	**	180.2 ± 36.3	120.5 ± 43.1
	3	ns	41.8 ± 13.8	32.4 ± 10.1	*	49.9 ± 12.2	38.8 ± 10.4	*	188.8 ± 56.6	131.8 ± 49.3
	4	ns	46.6 ± 14.7	37.9 ± 11.8	ns	39.5 ± 9.6	36.6 ± 8.5	*	160.1 ± 39.0	120.4 ± 41.4
**E** **F** **E**	1	ns	84.7 ± 12.3	78.5 ± 11.7	ns	44.6 ± 9.4	48.8 ± 10.7	ns	173.4 ± 44.5	150.6 ± 39.0
	2	ns	70.6 ± 11.6	66.6 ± 11.7	ns	60.4 ± 8.1	58.8 ± 8.2	ns	196.8 ± 30.6	174.5 ± 39.5
	3	ns	93.3 ± 12.9	84.0 ± 11.2	ns	84.6 ± 9.3	78.7 ± 11.8	*	281.1 ± 59.3	226.6 ± 63.3
	4	ns	84.7 ± 13.4	84.3 ± 11.6	ns	48.3 ± 6.0	53.5 ± 6.9	ns	227.7 ± 43.2	213.9 ± 43.6
**F** **P** **S**	1	ns	50.7 ± 21.5	50.2 ± 17.7	ns	31.0 ± 11.5	36.3 ± 11.2	ns	113.9 ± 24.3	125.4 ± 37.1
	2	ns	36.7 ± 19.5	43.2 ± 18.6	**	46.9 ± 12.6	62.9 ± 15.0	ns	182.4 ± 44.5	190.5 ± 69.2
	3	ns	49.7 ± 22.2	38.8 ± 19.9	ns	64.2 ± 11.5	62.5 ± 17.9	ns	196.2 ± 49.1	154.7 ± 52.7
	4	ns	43.3 ± 21.0	45.1 ± 19.8	ns	46.6 ± 9.7	56.6 ± 15.0	ns	188.6 ± 67.7	184.0 ± 50.4
**W** **F** **E**	1	**	35.6 ± 11.4	22.8 ± 10.5	ns	74.2 ± 14.4	81.0 ± 16.4	ns	283.1 ± 74.0	259.6 ± 68.2
	2	*	28.4 ± 13.6	14.8 ± 10.2	ns	57.2 ± 7.4	55.2 ± 11.5	ns	276.5 ± 78.2	219.9 ± 87.4
	3	*	0.9 ± 14.9	-12.7 ± 10.5	ns	34.6 ± 10.9	41.9 ± 11.1	ns	162.9 ± 65.2	138.2 ± 37.0
	4	**	44.5 ± 13.6	28.6 ± 10.9	ns	61.7 ± 10.1	58.6 ± 13.1	*	299.9 ± 63.0	237.5 ± 65.5
**W** **U** **R** **D**	1	ns	24.6 ± 11.4	24.3 ± 7.5	**	37.7 ± 8.5	26.4 ± 8.2	**	134.9 ± 34.7	81.9 ± 26.4
	2	ns	23.6 ± 9.6	24.2 ± 7.6	ns	27.7 ± 6.1	23.1 ± 7.7	**	122.5 ± 35.3	84.1 ± 26.9
	3	ns	15.8 ± 7.4	18.1 ± 8.2	ns	25.1 ± 6.2	20.6 ± 6.3	**	115.0 ± 35.4	73.7 ± 22.1
	4	ns	26.9 ± 11.7	26.5 ± 7.8	ns	23.5 ± 6.0	20.5 ± 8.8	*	126.4 ± 33.9	91.8 ± 34.6

Angular kinematic values include peak angle (degrees), range of motion (degrees), and peak angular velocity (degrees/s) of each movement (M) for trunk flexion/extension (TFE), lateral bending (TLB), and axial rotation (TAR); shoulder flexion/extension (SFE), abduction/adduction (SAA), and internal/external rotation (SIER); elbow flexion/extension (EFE) and forearm pronation/supination (FPS); and wrist flexion/extension (WFE) and ulnar/radial deviation (WURD). For the results of the pairwise comparisons (in column *p*),

* indicates a *p* value less than 0.05,

** indicates a *p* value less than 0.005, and

ns indicates a *p* value that is not significant.

Highlighted table cells also indicate significant differences (red = higher and blue = lower repeated study value).

The original- and repeated-study participants also had similar ROM values in Pasta, although significant differences were found for the Movement 2 trunk flexion/extension ROM and the Movement 2 & 3 trunk lateral bending ROM. However, these differences were quite small (with the largest being 5.3°). In Cups, differences in ROMs were significant in more movements and degrees of freedom (DOFs), as indicated by the shading in Table 5. However, the significant trunk ROM differences were quite small (both less than 2°), and the significant shoulder ROM differences were less than the respective original study standard deviations for those DOFs.

The repeated-study participants exhibited differences in peak angular velocities in most DOFs in both tasks. The peak angular velocities in the trunk DOFs of repeated-study participants were usually greater than those of original-study participants, with significant trunk flexion/extension differences in Movement 1 and 2 of Pasta and Movement 1 of Cups. The peak angular velocities in the remaining DOFs of the repeated-study participants were usually smaller than for the original-study participants, with most significantly lower.

### Eye gaze

The repeated- and original-study participants exhibited similar eye fixations, with no significant differences identified in either task, as shown in Table 6 (Pasta) and Table 7 (Cups). Significant eye arrival latency differences were identified in all Grasp phase transitions and the Movement 3 Release phase transition of Pasta, as well as the Movement 3 phase transitions of Cups. No significant eye leaving latency differences were identified in Pasta, but significant differences were identified in the Movement 3 Release transition in Cups.

**Table 6:** Pasta Box Task eye movement values (presented as means ± between-participant standard deviations), with significant results of the pairwise comparisons.

		Percent Fixation to Current (%)			Number of Fixations to Current		
Movement	Phase	*p*	Original	Repeated	*p*	Original	Repeated
1	Reach	ns	42.8 ± 8.6	49.4 ± 13.6	ns	1.01 ± 0.14	1.02 ± 0.05
	Grasp	ns	82.7 ± 15.0	73.9 ± 20.4	ns	0.97 ± 0.07	0.98 ± 0.04
	Transport	ns	75.1 ± 9.7	78.3 ± 9.1	ns	1.03 ± 0.05	1.05 ± 0.07
	Release	ns	71.8 ± 18.0	72.7 ± 25.1	ns	0.99 ± 0.03	1.02 ± 0.15
2	Reach	ns	77.5 ± 15.0	86.4 ± 14.9	ns	1.00 ± 0.02	1.02 ± 0.08
	Grasp	ns	89.4 ± 15.3	81.3 ± 19.9	ns	0.93 ± 0.15	0.90 ± 0.21
	Transport	ns	76.9 ± 9.3	81.0 ± 10.8	ns	1.02 ± 0.06	1.05 ± 0.06
	Release	ns	81.8 ± 15.1	81.5 ± 18.7	ns	0.99 ± 0.03	1.05 ± 0.21
3	Reach	ns	66.4 ± 15.8	80.4 ± 17.5	ns	1.02 ± 0.04	1.05 ± 0.13
	Grasp	ns	93.6 ± 14.0	91.6 ± 13.4	ns	0.98 ± 0.05	1.02 ± 0.08
	Transport	ns	50.0 ± 4.7	54.1 ± 8.7	ns	1.06 ± 0.09	1.10 ± 0.08
	Release	ns	64.2 ± 15.8	63.2 ± 20.0	ns	1.00 ± 0.09	0.97 ± 0.16
		Percent Fixation to Hand Only (%)			Number of Fixations to Hand Only		
Movement	Phase	*p*	Original	Repeated	*p*	Original	Repeated
1	Reach	ns		14.5 ± 1.8	ns	0.00 ± 0.00	0.03 ± 0.08
	Transport	ns	5.8 ± 2.4	9.3 ± 6.4	ns	0.32 ± 0.33	0.29 ± 0.29
2	Reach	ns	10.5 ± 9.0	5.1 ± 0.0	ns	0.03 ± 0.10	0.01 ± 0.05
	Transport	ns	12.7 ± 6.7	11.0 ± 6.5	ns	0.75 ± 0.28	0.64 ± 0.38
3	Reach	ns	10.5 ± 7.7	6.2 ± 1.9	ns	0.07 ± 0.19	0.09 ± 0.16
	Transport	ns	11.2 ± 3.6	10.9 ± 3.7	ns	0.85 ± 0.27	0.83 ± 0.26
		Eye Arrival Latency (seconds)			Eye Leaving Latency (seconds)		
Movement	Transition	*p*	Original	Repeated	*p*	Original	Repeated
1	Grasp	*	0.55 ± 0.11	0.79 ± 0.28	ns	0.02 ± 0.08	0.01 ± 0.15
	Release	ns	0.82 ± 0.20	1.04 ± 0.26	ns	-0.30 ± 0.20	-0.55 ± 0.64
2	Grasp	*	0.58 ± 0.14	0.82 ± 0.28	ns	-0.09 ± 0.13	-0.06 ± 0.16
	Release	ns	0.87 ± 0.17	1.1 ± 0.31	ns	-0.34 ± 0.19	-0.67 ± 0.67
3	Grasp	*	0.62 ± 0.17	0.91 ± 0.36	ns	-0.12 ± 0.09	-0.14 ± 0.15
	Release	*	0.66 ± 0.10	0.90 ± 0.25	ns	-0.23 ± 0.09	-0.34 ± 0.25

For the results of the pairwise comparisons (in column p),

* indicates a significant p value less than 0.05 and

“ns” indicates a p value that is not significant.

Highlighted table cells also indicate significant differences (red = higher repeated study value).

**Table 7:** Cup Transfer Task eye movement values (presented as means ± between-participant standard deviations), with significant results of the pairwise comparisons.

		Percent Fixation to Current (%)			Number of Fixations to Current		
Movement	Phase	*p*	Original	Repeated	*p*	Original	Repeated
1	Reach	ns	71.8 ± 14.6	68.3 ± 16.8	ns	1.00 ± 0.11	0.89 ± 0.25
	Grasp	ns	82.5 ± 21.3	85.6 ± 15.3	ns	0.89 ± 0.16	0.87 ± 0.20
	Transport	ns	78.8 ± 11.4	70.0 ± 16.1	ns	1.02 ± 0.03	0.97 ± 0.27
	Release	ns	58.0 ± 18.2	67.2 ± 15.1	ns	0.87 ± 0.18	0.90 ± 0.25
2	Reach	ns	92.6 ± 7.4	87.6 ± 11.0	ns	1.01 ± 0.02	1.01 ± 0.26
	Grasp	ns	86.7 ± 13.9	89.2 ± 13.6	ns	0.95 ± 0.10	0.94 ± 0.27
	Transport	ns	79.5 ± 11.9	72.0 ± 9.8	ns	1.01 ± 0.02	1.08 ± 0.08
	Release	ns	82.2 ± 16.7	90.1 ± 8.3	ns	0.94 ± 0.22	1.02 ± 0.06
3	Reach	ns	77.6 ± 15.3	75.3 ± 18.0	ns	1.00 ± 0.07	1.14 ± 0.17
	Grasp	ns	90.4 ± 14.5	92.8 ± 12.3	ns	0.90 ± 0.24	0.97 ± 0.12
	Transport	ns	74.5 ± 10.7	70.0 ± 10.9	ns	1.03 ± 0.05	1.06 ± 0.13
	Release	ns	75.7 ± 15.3	81.3 ± 16.0	ns	0.94 ± 0.15	0.98 ± 0.10
4	Reach	ns	85.3 ± 12.1	71.9 ± 20.6	ns	0.96 ± 0.09	0.84 ± 0.30
	Grasp	ns	78.1 ± 21.9	83.0 ± 21.4	ns	0.83 ± 0.27	0.86 ± 0.23
	Transport	ns	66.2 ± 14.6	65.2 ± 9.7	ns	0.96 ± 0.14	1.00 ± 0.14
	Release	ns	82.5 ± 18.9	86.4 ± 17.2	ns	0.94 ± 0.17	0.95 ± 0.20
		Percent Fixation to Hand Only (%)			Number of Fixations to Hand Only		
Movement	Phase	*p*	Original	Repeated	*p*	Original	Repeated
1	Reach	ns	14.3 ± 6.7	12.0 ± 7.3	ns	0.02 ± 0.04	0.08 ± 0.12
	Transport	ns	8.2 ± 4.8	14.0 ± 9.5	ns	0.52 ± 0.32	0.73 ± 0.47
2	Reach	ns	7.5 ± 4.2	7.5 ± 5.0	ns	0.08 ± 0.09	0.08 ± 0.13
	Transport	ns	10.3 ± 4.0	14.8 ± 6.8	ns	0.58 ± 0.29	0.75 ± 0.34
3	Reach	ns	7.3 ± 4.8	6.3 ± 4.0	ns	0.07 ± 0.17	0.05 ± 0.15
	Transport	ns	11.8 ± 6.1	14.9 ± 6.4	ns	0.74 ± 0.33	0.89 ± 0.32
4	Reach	ns	16.8 ± 12.2	15.8 ± 11.8	ns	0.11 ± 0.18	0.23 ± 0.34
	Transport	ns	13.7 ± 9.6	17.3 ± 4.4	ns	0.68 ± 0.43	0.85 ± 0.33
		Eye Arrival Latency (seconds)			Eye Leaving Latency (seconds)		
Movement	Transition	*p*	Original	Repeated	*p*	Original	Repeated
1	Grasp	ns	0.66 ± 0.16	0.75 ± 0.39	ns	-0.02 ± 0.10	-0.14 ± 0.24
	Release	ns	0.82 ± 0.15	0.90 ± 0.23	ns	-0.15 ± 0.10	-0.30 ± 0.18
2	Grasp	ns	0.73 ± 0.16	0.86 ± 0.35	ns	-0.04 ± 0.10	-0.18 ± 0.23
	Release	ns	0.94 ± 0.12	1.04 ± 0.21	ns	-0.32 ± 0.26	-0.58 ± 0.30
3	Grasp	*	0.93 ± 0.19	1.21 ± 0.36	ns	-0.06 ± 0.18	-0.18 ± 0.16
	Release	*	0.87 ± 0.16	0.98 ± 0.22	*	-0.22 ± 0.11	-0.41 ± 0.19
4	Grasp	ns	0.57 ± 0.10	0.61 ± 0.33	ns	-0.06 ± 0.19	-0.17 ± 0.17
	Release	ns	0.70 ± 0.17	0.82 ± 0.16	ns	-0.34 ± 0.19	-0.53 ± 0.28

For the results of the pairwise comparisons (in column p),

* indicates a significant p value less than 0.05 and

“ns” indicates a p value that is not significant.

Highlighted table cells also indicate significant differences (red = higher and blue = lower repeated study value).

## Discussion

Measures that were consistent between the original and repeated studies included all hand velocity, grip aperture, and eye fixation results, along with most peak joint angle and ROM results. Although participants in the repeated study took more time to complete each functional task (greater overall duration), similar relative phase durations between the participant groups indicated that the repeated-study participants did not spend a disproportionate amount of time in any one phase.

Participants in the original study may have displayed faster performance due to the prior functional task trials that they completed (that is, during task trials where only motion capture or eye tracking data were captured in the original study). This presumption is likely, given that practice has been shown to decrease functional test completion time [24]. The longer phase durations exhibited by the repeated-study participants directly affected the eye arrival latencies and eye leaving latencies due to the time-dependent nature of these calculations. Furthermore, the longer movement times resulted in decreased joint angular velocities in shoulder, elbow, forearm, and wrist DOFs.

Learning effects may have also contributed to discrepancies in hand movement measures between the original- and repeated-study participants. The repeated-study participants exhibited an increased number of movement units and increased hand trajectory variability, both of which were likely due to the influence of fewer practice opportunities [25,26]. Furthermore, increased hand trajectory variability presumably contributed to the increased average hand distance travelled of the repeated-study participants. Hand trajectory variances would be expected to be away from, or in avoidance of, obstacles present in all task movements (box walls and the partition in the Cup Transfer Task, and the shelf frames in the Pasta Box Task). Future studies that employ GaMA should standardize the amount of functional task practice opportunities that participants receive.

Task demonstration variations by raters may also have contributed to task duration differences between the two participant groups. Although the same script was used to explain the tasks to participants in each study, small variances in task demonstration speed may have been introduced by the raters. Since the timing of demonstrations is known to influence the resulting pace of participants’ movements [27], a slower demonstration may have contributed to the repeated study’s increase in task duration time. It is recommended that a standard task demonstration video be created and shown to all participants to reduce the possible effects of rater demonstration variation.

The angular kinematic measures revealed offsets in the wrist flexion/extension and ulnar/radial deviation measures of the repeated-study participants, likely due to differences in the kinematic calibration pose across the two studies. Such calibration errors are known to be the main limitation of the *Clusters Only* model [20]. In addition, a large standard deviation in trunk flexion/extension was observed for repeated-study participants, also likely attributable to errors in the kinematic calibration. That is, the calibration of this DOF depends on how each participant chooses to ‘stand upright’. To limit such deviations in joint angles, the rater must ensure that the participant does not have a bent wrist and is standing as upright as possible, when a kinematic calibration pose is captured.

Further angular kinematics variations were observed between the two participant groups, in both the forearm pronation/supination and wrist ulnar/radial deviation ROMs. Such deviations were introduced by the *Clusters Only* model, which calculates wrist and forearm angles in a manner that is different from other DOFs. This alternative calculation method was chosen because, during the required calibration pose, participants struggled to align their wrist axes of rotation with the global coordinate system, either due to their elbow carrying angle or their inability to supinate their forearm the required amount. As such, the model uses the local coordinate system of the forearm plate to calculate wrist and forearm joint angles. Small misplacements of the forearm marker plate, however, can introduce wrist and forearm joint angle calculation errors. To combat this limitation of the *Clusters Only* model, the rater must take care to align the forearm marker plate with the long axis of the forearm when it is affixed to the participant. A second option would be to use a calibration model with both marker clusters and anatomical markers, which may increase accuracy specifically for wrist and forearm kinematics [20].

Although little has been done to validate eye tracking and/or motion capture methods in upper limb movement research, many studies have validated motion capture methods for gait measurements [28]. Gait studies commonly revealed that inconsistencies in motion capture marker placement were a large source of anatomical model errors [28]. The *Clusters Only* model used by GaMA attempts to address this issue as it does not require precise individual marker placement, and has been shown to be as reliable as an anatomical model [20]; it does, however, introduce its own variability caused by calibration pose inconsistencies. Gait reliability research has also identified intrinsic participant-to-participant variation within a given population and trial-to-trial variation for a given participant [28,29]. Such variation could, at least partially, also explain movement behaviour differences between the original and repeated data sets of this study.

### Limitations

Given that this study manipulated numerous experimental factors when comparing the visual and movement measures of two groups of non-disabled participants, it had limitations. It was infeasible for this research to determine the degree to which these factors (different participants, sites, equipment, raters, and task experience opportunities) affected movement measure variation. Additional research on the effects of training could shed more light onto whether or not the amount of practice fully explains the difference in results between the two studies. Although assessment of inter-site/inter-rater reliability of GaMA using the same participant group would also provide valuable information by reducing the effects of inter-participant variability, for this study, a new participant group presented an opportunity to analyze a wider range of normative behaviour; an important consideration when designing an assessment tool to be used to characterize functional impairments.

## Conclusions

Overall, the results of the repeated study were similar to those obtained by Valevicius et al. and Lavoie et al. [7–9]. Most hand movement, angular joint kinematic, and eye gaze results exhibited by participants in the repeated study were consistent with those observed in the original study. Most significant differences between the results could be explained by the amount of practice that participants in the two studies received, demonstration variations introduced by the rater, and the limitations of the *Clusters Only* kinematics model. Researchers should be aware of such potential variability when collecting data, and endeavor to adhere to the same data collection protocol when intending to compare data across sites. GaMA presents a novel methodology to obtain quantitative metrics on hand function, trunk and angular joint kinematics, along with eye fixation behaviour during functional object manipulation tasks. Due to its demonstrated reproducibility, it is expected that, in the future, GaMA can serve as a reliable and informative functional assessment tool across different sites and for individuals with sensorimotor impairments in the upper limb.

## Supporting information

S1 Text Additional Details regarding the Gaze and Movement Assessment (GaMA). (DOCX)

S2 Text Detailed statistical analysis. (DOCX)

S1 Table Phase duration values for the Pasta Box Task and Cup Transfer Task (presented as means ± between-participant standard deviations), with significant results of the pairwise comparisons. For the results of the pairwise comparisons (in column p), * indicates a significant p value less than 0.05, ** indicates a p value less than 0.005, and “ns” indicates a p value that is not significant.
(DOCX)

S2 Table Pasta Box Task hand movement values for each movement segment (presented as means ± between-participant standard deviations), with significant results of the pairwise comparisons. For the results of the pairwise comparisons (in column p), * indicates a significant p value less than 0.05, ** indicates a p value less than 0.005, and “ns” indicates a p value that is not significant.
(DOCX)

S3 Table Cup Transfer Task hand movement values for each movement segment (presented as means ± between-participant standard deviations), with significant results of the pairwise comparisons. For the results of the pairwise comparisons (in column p), * indicates a significant p value less than 0.05, ** indicates a p value less than 0.005, and “ns” indicates a p value that is not significant.
(DOCX)
