# Supplementary material for: Gaze and Movement Assessment (GaMA): Inter-site validation of a visuomotor upper limb functional protocol
Source: PLoS One. 2019 Dec 30;14(12):e0219333. doi: 10.1371/journal.pone.0219333 (PMC6936776; doi:10.1371/journal.pone.0219333)
Supplement: S1 Text — (DOCX) [file pone.0219333.s001.docx]

# Supplement 1: Additional Details regarding the Gaze and Movement Assessment (GaMA)

This supplement provides additional details about the Gaze and Movement Assessment (GaMA) protocol. GaMA encompasses the following:

1. A protocol for the administration of two functional tasks that incorporate common dextrous hand demands of daily living – the ‘Pasta Box Task’ and ‘Cup Transfer Task’;
2. A methodology to use motion capture and eye tracking hardware and software solutions to acquire synchronized movement and eye data during functional task execution; and
3. Analysis software (available as an executable file), which calls for a standardized data set of synchronized movement and eye data coordinates as input, and outputs measures of hand movement, angular joint kinematics, and eye gaze

What follows is information about each of the abovementioned components of GaMA, including a justification for the methods employed, and the responsibilities of a rater (experimenter) tasked with the acquisition and preparation of movement and eye data.

# S1 – 1 GaMA’s Two Functional Tasks

GaMA’s two functional tasks, known as the ‘Pasta Box Task’ and the ‘Cup Transfer Task’, were developed by an interdisciplinary team through an iterative process based on the movement requirements of current functional assessment tasks. These tasks were designed to mimic activities of daily living and to be challenging, but not impossible, for clinical populations to perform [1]. They were also designed to include distinct, standardized movements that could easily be segmented into reach, grasp, transport, and release phases, allowing for detailed visuomotor analysis.

## The Pasta Box Task


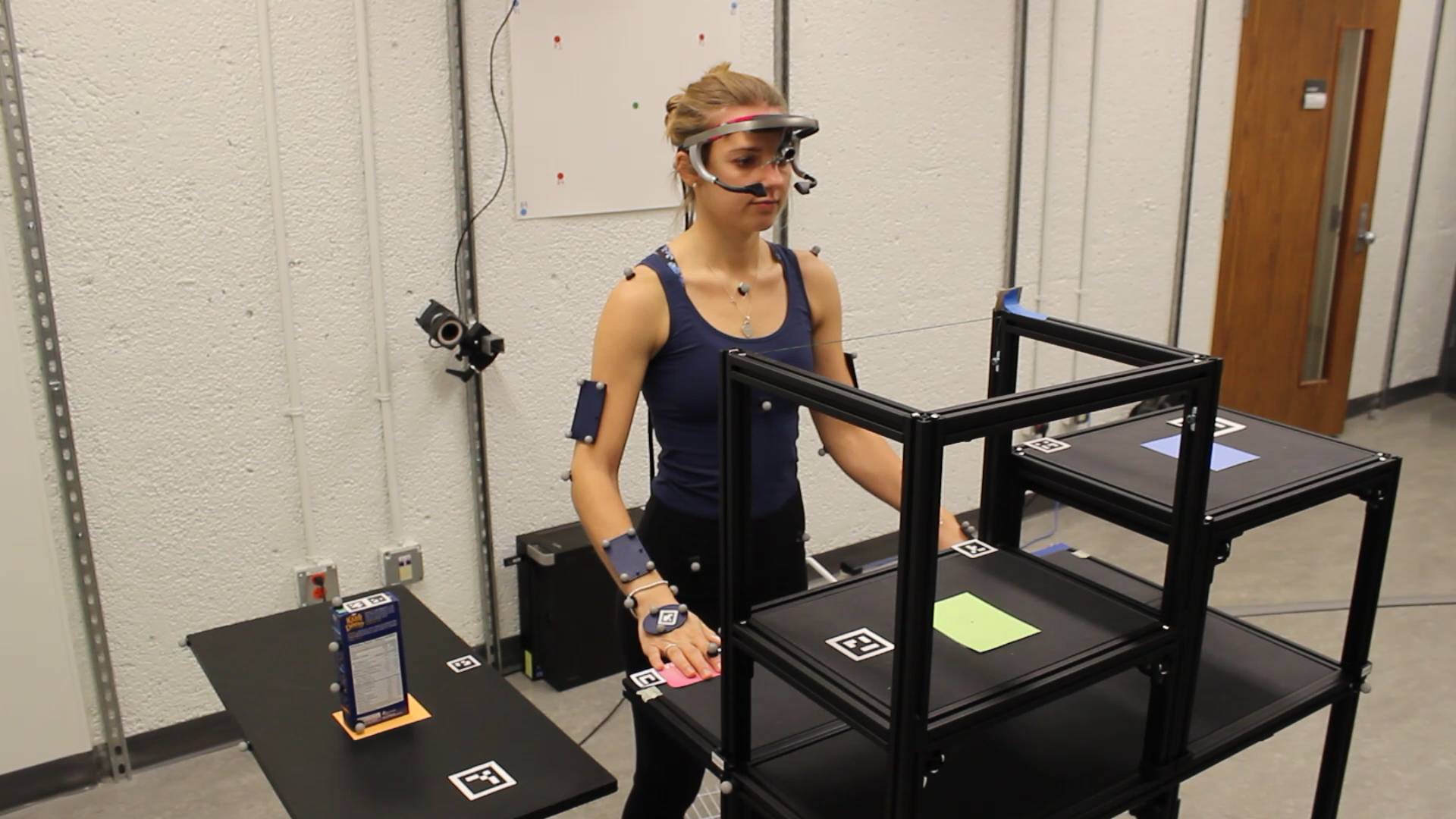


Figure S1-1: Pasta Box Task setup

The Pasta Box Task, shown in Figure S1-1, requires a research participant to move a deformable pasta box (specifically, a 225g box of Kraft Dinner Original) between a counter and shelves at different heights in front of them – a task that mimics reaching for a kitchen item and moving it to a counter or shelf. This task is made up of three distinct movements:

1. The participant reaches for and grasps a pasta box on a table beside them (on the side of the hand that they are using to perform the task), transports the box and releases it on a shelf in front of them, and then moves their hand to a standardized “home” position (necessary for data segmentation).
2. The participant reaches for and grasps the pasta box from the new location, transports it and releases it on a higher shelf across their midline, and then moves their hand back to the home position.
3. The participant reaches for and grasps the pasta box, transports it and releases it back on the original side table, and then moves their hand to the home position.

## The Cup Transfer Task


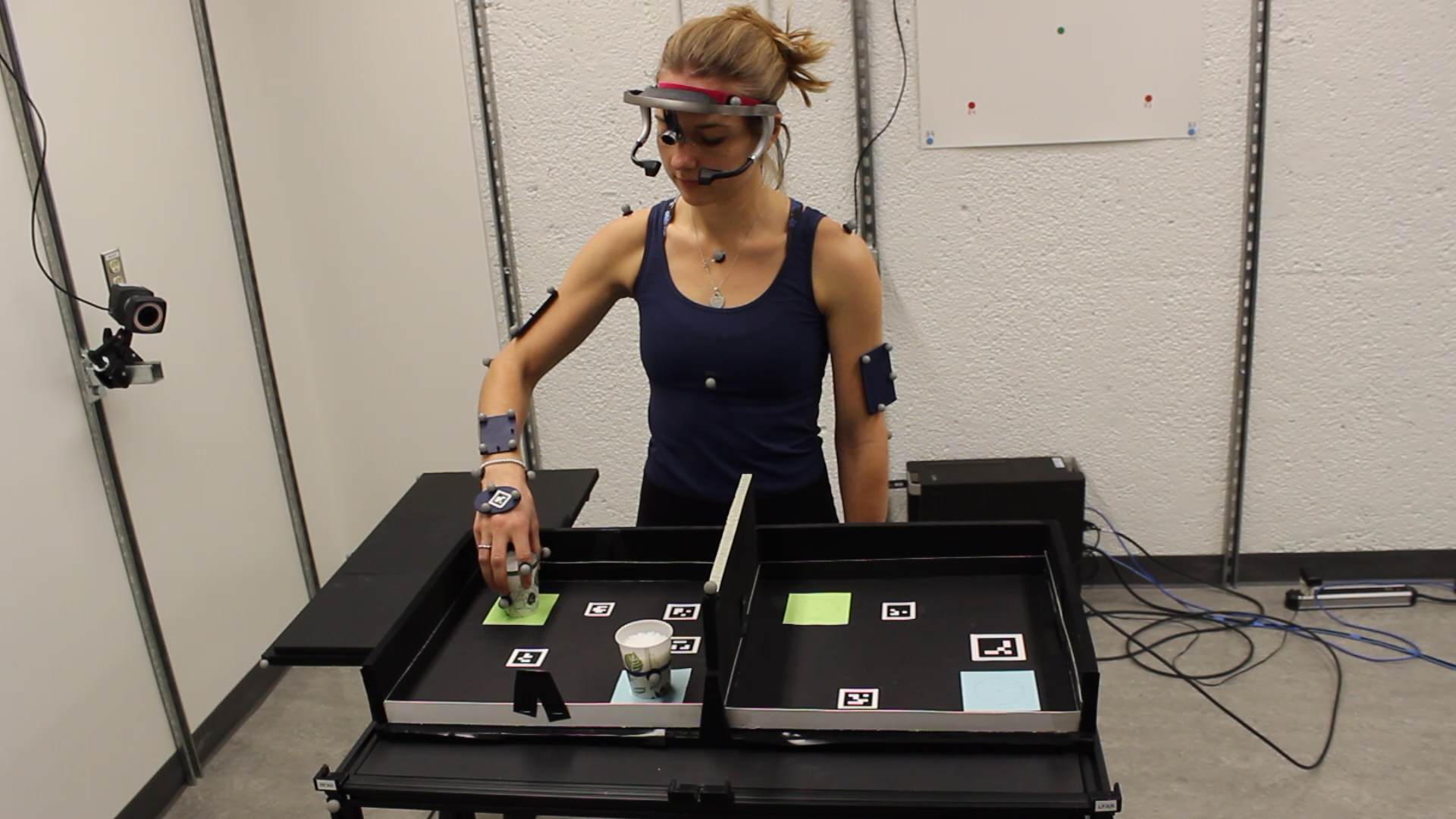


Figure S1-2: Cup Transfer Task setup

The Cup Transfer Task, shown in Figure S1-2, introduces greater risk by using deformable cups filled with beads (specifically, 5oz Dixie® Wax Treated Paper Cold Cups filled with soft plastic pellets), and requires a participant to carefully navigate the cup over a barrier before setting it down on the opposite side of the box – a task that mimics moving filled, open containers around a sink or countertop using different grasp patterns. The participant is also required to use different grasp patterns: top grip and side grip of the cup. This task involves four distinct movements:

1. The participant reaches for a ‘near’ cup (on the side of the testing arm), grasps it with a top grip, transports it across their midline over a barrier, and releases it on a specified target on the opposite side of the barrier.
2. The participant reaches for a ‘far’ cup, grasps it with a side grip, transports it across their midline over the barrier, releases it on a specified target, and then moves their hand to a home position.
3. The movements are then reversed, as the participant reaches for the ‘far’ cup on the opposite side of the testing arm, grasps it with a side grip, transports it back over the barrier, and releases it on its original target.
4. The participant lastly reaches for the “near” cup, which is on the opposite side from the testing arm, grasps it with a top grip, transports it back over the barrier, releases it on its original target, and then moves their hand to the home position.

To allow task setups to be replicable at other sites, each task’s specifications are defined in detail. For instance, the task cart, side table, and shelves all have specific heights and surface dimensions, the pasta box and cups have specific dimensions and weights, and task targets and positions have specific sizes and locations. Detailed task protocols were published by Valevicius et al. [1].

# S1 – 2 Acquisition of synchronized movement and eye data during functional task execution

GaMA’s data collection methodology is intended to be amenable to various eye tracking and motion capture technologies, given that such equipment will inevitably become more advanced. Regardless of the technologies employed, the end goal is to acquire the synchronized movement and eye data of a research participant executing GaMA’s functional task(s). Current data acquisition methods use optical motion capture equipment (with a “Clusters Only” motion capture model [2]) along with a head-mounted eye tracker. The “Clusters Only” model has been shown to be reliable, to minimize marker occlusion, and to maximize ease-of-use [2]. A head-mounted eye tracker allows participants to move their head freely while performing the functional tasks, as opposed to a desktop- or tower-mounted eye tracker, which would require participants to restrict their head movements.

The rater must attend to the following tasks to synchronously collect movement and eye data:

**Setup** – positioning of data collection materials and equipment, calibration of the motion capture system, attachment of motion capture markers/marker plates, and positioning of the eye tracker

**Calibration –** performing angular kinematics and eye gaze calibrations

**Task Explanation & Demonstration**

**Data Recording & Monitoring**

**Data Post-Processing** ­– cleaning data and reformatting data

## Setup

### Positioning of data collection materials and equipment

First, the rater must set up the data collection space, equipped with motion capture cameras positioned for data collection, a computer with the appropriate software installed for data capture, and the equipment necessary for the two functional tasks.

### Calibration of the motion capture system

The motion capture cameras must be calibrated by the rater so that the three-dimensional positions of motion capture markers can be calculated.

#### **Attachment of motion capture markers/marker plates**

Individual markers are to be affixed to the participants’ index finger (middle phalange) and thumb (distal phalange), and four individual markers are to be affixed to a headband worn by the participant. Motion capture marker plates are also to be affixed to the participant, as shown in Figure S1-3.


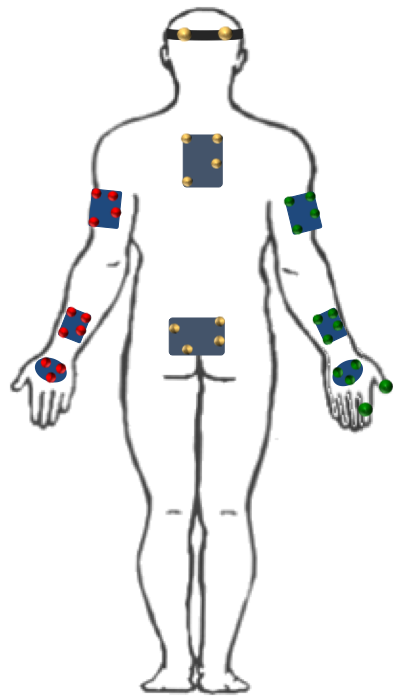


Figure S1-3: Marker placement on a participant

Marker plates are to be affixed as follows:

- 1. Posterior pelvis: on sacrum, just below a line connecting the two PSIS landmarks
  2. Posterior thorax (upper back): centered between shoulder blades (mid medial border of scapula from about T4-T8)
  3. Upper arms: middle of upper arm, lateral surface
  4. Forearms: dorsal forearm, just above styloids (to not interfere with wrist motion), along long axis of forearm. Ensure that the plate is high enough on the forearm so that no occlusion of hand or forearm markers occurs
  5. Hands: dorsum of hand

### Positioning of the eye tracker

The head-mounted eye tracker is to be placed on the participant and the eye camera(s) are to be positioned by the rater, such that the pupils are visible when the participants look up, down, right, and left. Additional settings (such as pupil intensity) can be adjusted, when available, to further improve eye tracking.

## Calibration

### Performing angular kinematics calibration

For the angular kinematics calibration, the participant is to stand in an anatomical position with no shoulder abduction (to define 0° joint angles for the “Clusters Only” model), as shown in Figure S1-4. The rater must ensure that the participant is standing in this position correctly (standing upright in front of the task cart with shoulders back, with elbow and wrist axes of rotation parallel to edge of task cart, and with wrists held straight with no extension). The participant must stand in this position for 3 calibration trials, each at least 2 seconds long. Motion capture data must be collected for these trials.


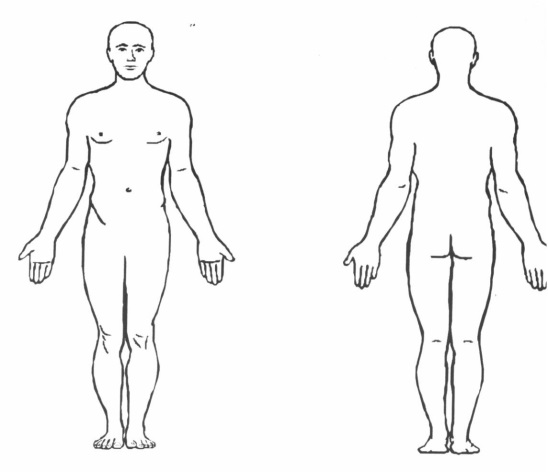


Figure S1-4: Anatomical position

### Performing eye gaze calibrations

Eye gaze vector calibrations are required to determine the virtual location of the participant’s gaze throughout the task [3]. The goal of the eye gaze vector calibration is to have the participant stare at the marker at the tip of the eye gaze vector calibration wand, depicted in Figure S1-5, as closely as possible. At least one eye gaze vector calibration should be performed before each task, and again at the end of the last task. The stationary gaze calibration is recommended, and requires the participant to move their head up, down, right, and left, and then two spirals (clockwise and counter-clockwise), all while maintaining visual focus on the marker at the tip of the eye gaze vector calibration wand. Motion capture and eye tracking data must be collected for these gaze calibration trials.


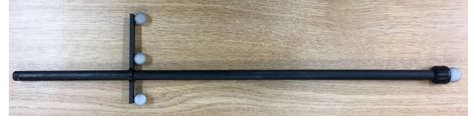


Figure S1-5: Eye gaze vector calibration wand

## Task Explanation & Demonstration

Raters should explain and demonstrate each task immediately before the participant performs trials of that particular task. Task instruction details are included in each task protocol [1].

## Data Recording & Monitoring

Synchronization of motion capture and eye tracking data depends on the specific technologies used. For example, if a Vicon motion capture system and Dikablis eye tracker are used, software triggers can be used to simultaneously start and stop recording from these two data capture technologies. On the other hand, if an OptiTrack motion capture system and Pupil Labs eye tracker are used, a third-party software solution, such as LabStreamingLayer, can be used to collect pupil position timestamps and motion capture timestamps, which can be used to align data after collection. The rater must ensure that data is recorded accurately as participants perform the tasks. The rater must ensure that motion capture markers are visible, with limited occlusion, and that motion capture markers/plates do not fall off or move. The rater must also ensure that the eye tracker headset or eye cameras do not move after eye gaze calibration is performed.

## Data Post-Processing

### Data Cleaning

Before motion capture and eye tracking data can be analyzed using the GaMA software, the data must be cleaned and synchronized. Motion capture data must be cleaned in the corresponding motion capture software. Doing so ensures that: marker position data are labelled correctly; gaps in marker positions (introduced by marker occlusion) are filled using appropriate interpolation methods; and noisy marker position data are filtered using a second-order, low-pass Butterworth filter with a cut-off frequency of 6 Hz. Eye tracking data, on the other hand, can be cleaned automatically using custom Matlab scripts. Eye data cleaning involves: removing erroneous pupil position data; filling gaps in data (introduced by blinking) using interpolation; and filtering noisy data using a second-order, low-pass Butterworth filter with a cut-off frequency of 10 Hz.

#### **Data Reformatting**

Finally, these data streams must be aligned, based on the synchronization solution used, and then the data can be reformatted for the GaMA software. The comma-separated value (CSV) files that are read by GaMA must contain time stamps, frame numbers, pupil x- and y-positions (one column for x, one for y), and motion capture x-, y-, and z-positions of each marker (three columns for each marker).

# S1 – 3 Analysis Software

GaMA software calls for a standardized data set of synchronized movement and eye data coordinates as input, and outputs measures of hand movement, angular joint kinematics, and eye gaze. With the required data input, this software provides mechanisms for the rater to achieve the following:

1. Virtual objects can be created to represent the participant’s head, hand, and task areas of interest (AOIs)
   1. To do this, first rigid bodies can be created for the head, hand, cart, side table, and pasta box
   2. Rigid bodies or individual markers are then used to create virtual objects
2. The gaze vector can then be calculated
   1. To accomplish this, the GaMA software can be used to create a regression function using the x- and y-coordinates of each eye collected by the eye tracker, the motion capture markers on the participant’s head, and the motion capture marker on the tip of the eye gaze vector calibration wand from the eye gaze vector calibration trials
   2. This regression function can then be applied to the task trial data, yielding a virtual location of the participant’s gaze (as represented by a gaze vector)
3. Gaze fixations to AOIs can be detected
   1. Fixations are considered to be a continuous glance to an AOI by the participant that is longer than 100 milliseconds and within a given distance tolerance (tolerances outlined by Lavoie et al. [3])

**Note:** Configuration files can be saved and batch-applied to synchronized data files to complete steps 1-3 for multiple files using identical parameters.

1. GaMA analysis software then can be used to segment trials into reach, grasp, transport, and release phases of each distinct movement (the parameters used for this are outlined by Valevicius et al. [1])
2. Finally, GaMA software can calculate all visuomotor outcome measures of interest, including those of hand movement, angular joint kinematics, and eye gaze. These outcome measures are saved as Matlab MAT files.

# References for Supplement 1

[1] A. M. Valevicius *et al.*, “Characterization of normative hand movements during two functional upper limb tasks,” *PLoS One*, vol. 13, no. 6, p. e0199549, 2018.

[2] Q. A. Boser *et al.*, “Cluster-based upper body marker models for three-dimensional kinematic analysis: Comparison with an anatomical model and reliability analysis,” *J. Biomech.*, vol. 72, pp. 228–234, 2018.

[3] E. B. Lavoie *et al.*, “Using synchronized eye and motion tracking to determine high-precision eye-movement patterns during object-interaction tasks,” *J. Vis.*, vol. 18, no. 6, p. 18, 2018.
