# Supplementary material for: Gaze and Movement Assessment (GaMA): Inter-site validation of a visuomotor upper limb functional protocol
Source: PLoS One. 2019 Dec 30;14(12):e0219333. doi: 10.1371/journal.pone.0219333 (PMC6936776; doi:10.1371/journal.pone.0219333)
Supplement: S2 Text — (DOCX) [file pone.0219333.s002.docx]

To investigate differences between the two groups of participants, a series of repeated-measures analyses of variance (RMANOVAs) and pairwise comparisons were conducted for each measure and task. The designs of the initial RMANOVAs for each measure, which depended on the movement subsets (phases, movement segments, or phase transitions) for which each measure was analyzed, are outlined in Table S2-1.

Table S2-1: Movement subsets used for analysis of each measure, as well as initial RMANOVA design for each measure.

|  | | Measures | | | | | |
| --- | --- | --- | --- | --- | --- | --- | --- |
|  |  | Duration & Eye Movement | | | Hand Movement | | Angular Kinematics |
|  |  | Phase Duration  Relative Phase Duration  Percent Fixation to Current  Number of Fixations to Current | Percent Fixation to Hand  Number of Fixations to Hand | Eye Arrival Latency  Eye Leaving Latency | Hand Distance Travelled  Hand Trajectory Variability  Number of Movement Units  Peak Hand Velocity  Percent to Peak Hand Velocity | Peak Grip Aperture  Percent to Peak Grip Aperture  Percent to Peak Hand Deceleration | Peak Joint Angles  Joint Ranges of Motion  Peak Joint Angular Velocities |
| Movement Subsets | Reach | ✓ | ✓ |  |  |  |  |
|  | Grasp | ✓ |  |  |  |  |  |
|  | Transport | ✓ | ✓ |  |  |  |  |
|  | Release | ✓ |  |  |  |  |  |
|  | Reach-Grasp |  |  |  | ✓ | ✓ |  |
|  | Transport-Release |  |  |  | ✓ |  |  |
|  | End of Grasp |  |  | ✓ |  |  |  |
|  | Beginning of Release |  |  | ✓ |  |  |  |
|  | Movements Only |  |  |  |  |  | ✓ |
| Pasta Box Transfer Task Initial RMANOVA Design  (Group × Movement × Movement Subset) | | 2×3×4 | 2×3×2 | 2×3×2 | 2×3×2 | 2×3 | 2×3 |
| Cup Transfer Task Initial RMANOVA Design  (Group × Movement × Movement Subset) | | 2×4×4 | 2×4×2 | 2×4×2 | 2×4×2 | 2×4 | 2×4 |

The measures outlined in Table S2-1 were categorized as either: (1) measures where the initial RMANOVA had three factors and (2) measures where the initial RMANOVA had two factors. Details of analyses for each category follow, whereby all RMANOVA main effects or interactions were considered to be significant if the Greenhouse-Geisser corrected *p* value was less than 0.05, and all pairwise comparison results were considered to be significant if the Bonferroni corrected *p* value was less than 0.05. Furthermore, only significant group effects or interactions involving group were further investigated.

**Category 1 Measures:** If the initial three-factor RMANOVA indicated that a three-way interaction between group, movement, and movement subset was significant, then two-factor RMANOVAs were carried out for each movement subset with a 2 (group) × 3 (movement) design for the Pasta Box Transfer Task or a 2 (group) × 4 (movement) design for the Cup Transfer Task. Significant main effects or interactions (involving group) indicated by these two-factor RMANOVAs were followed up with pairwise comparisons between the two groups.

If the initial three-factor RMANOVA did not indicate a significant three-way interaction, two-way interactions between group and movement or movement subset were investigated. If such an interaction was found to be significant, then this interaction was followed up with a collapsed two-factor RMANOVA. Significant main effects or interactions (involving group) indicated by these two-factor RMANOVAs were further investigated using pairwise comparisons between the two groups.

If the initial three-factor RMANOVA did not indicate any significant interactions involving group, but did indicate a group main effect, then pairwise comparisons between the two groups were conducted for each combination of movement and movement subset.

**Category 2 Measures:** Significant group main effects or interactions between group and movement indicated by the initial RMANOVA were further investigated using pairwise comparisons between the two groups.
