# Supplementary material for: Gaze and Movement Assessment (GaMA): Inter-site validation of a visuomotor upper limb functional protocol
Source: PLoS One. 2019 Dec 30;14(12):e0219333. doi: 10.1371/journal.pone.0219333 (PMC6936776; doi:10.1371/journal.pone.0219333)
Supplement: S1 Table — For the results of the pairwise comparisons (in column p), * indicates a significant p value less than 0.05, ** indicates a p value less than 0.005, and “ns” indicates a p value that is not significant. (DOCX) [file pone.0219333.s003.docx]

Table S1: Phase duration values for the Pasta Box Task and Cup Transfer Task (presented as means ± between-participant standard deviations), with significant results of the pairwise comparisons. For the results of the pairwise comparisons (in column p), * indicates a significant p value less than 0.05, ** indicates a p value less than 0.005, and “ns” indicates a p value that is not significant.

| **Pasta Box Task** | | Duration (seconds) | | | Relative Duration (%) | | |  |
| --- | --- | --- | --- | --- | --- | --- | --- | --- |
| Movement | Phase | *p* | Original | Repeated | *p* | Original | Repeated |  |
| 1 | Reach | ns | 0.66 ± 0.08 | 0.78 ± 0.18 | ns | 29.03 ± 2.01 | 27.48 ± 3.36 |  |
|  | Grasp | * | 0.27 ± 0.08 | 0.40 ± 0.16 | ns | 11.47 ± 2.47 | 13.33 ± 2.89 |  |
|  | Transport | * | 1.08 ± 0.12 | 1.34 ± 0.33 | ns | 47.13 ± 2.22 | 46.73 ± 2.09 |  |
|  | Release | ns | 0.28 ± 0.07 | 0.37 ± 0.15 | ns | 12.37 ± 2.34 | 12.47 ± 2.45 |  |
| 2 | Reach | ns | 0.52 ± 0.06 | 0.61 ± 0.15 | ns | 24.44 ± 2.01 | 22.97 ± 2.21 |  |
|  | Grasp | * | 0.18 ± 0.05 | 0.28 ± 0.11 | ns | 8.32 ± 1.67 | 9.95 ± 2.06 |  |
|  | Transport | * | 1.12 ± 0.13 | 1.36 ± 0.32 | ns | 53.00 ± 2.89 | 51.03 ± 2.72 |  |
|  | Release | * | 0.30 ± 0.08 | 0.44 ± 0.18 | ns | 14.24 ± 2.73 | 16.06 ± 2.76 |  |
| 3 | Reach | ns | 0.65 ± 0.10 | 0.76 ± 0.18 | ns | 26.18 ± 1.82 | 24.78 ± 1.91 |  |
|  | Grasp | * | 0.19 ± 0.06 | 0.28 ± 0.12 | ns | 7.36 ± 1.78 | 8.57 ± 2.30 |  |
|  | Transport | * | 1.31 ± 0.16 | 1.60 ± 0.34 | ns | 52.91 ± 2.07 | 52.37 ± 3.57 |  |
|  | Release | ns | 0.34 ± 0.07 | 0.46 ± 0.19 | ns | 13.56 ± 2.16 | 14.73 ± 3.10 |  |
| **Cup Transfer Task** | | Duration (seconds) | | | Relative Duration (%) | | | |
| Movement | Phase | *p* | Original | Repeated | *p* | Original | Repeated |  |
| 1 | Reach | * | 0.66 ± 0.09 | 0.81 ± 0.19 | ns | 30.79 ± 1.72 | 29.04 ± 2.64 |  |
|  | Grasp | ** | 0.18 ± 0.05 | 0.28 ± 0.10 | ns | 8.38 ± 1.83 | 9.63 ± 2.17 |  |
|  | Transport | ** | 1.02 ± 0.10 | 1.23 ± 0.22 | ns | 47.77 ± 2.42 | 45.00 ± 4.68 |  |
|  | Release | ** | 0.28 ± 0.07 | 0.46 ± 0.14 | ns | 13.06 ± 2.34 | 16.34 ± 3.92 |  |
| 2 | Reach | * | 0.53 ± 0.09 | 0.66 ± 0.14 | ns | 24.00 ± 1.67 | 23.00 ± 2.43 |  |
|  | Grasp | * | 0.23 ± 0.07 | 0.32 ± 0.09 | ns | 10.26 ± 1.92 | 11.04 ± 1.92 |  |
|  | Transport | ** | 1.15 ± 0.12 | 1.42 ± 0.20 | ns | 52.15 ± 2.72 | 49.94 ± 4.42 |  |
|  | Release | ** | 0.30 ± 0.07 | 0.47 ± 0.16 | ns | 13.59 ± 2.88 | 16.01 ± 4.20 |  |
| 3 | Reach | ** | 0.88 ± 0.12 | 1.10 ± 0.24 | ns | 34.43 ± 2.03 | 33.62 ± 2.72 |  |
|  | Grasp | ** | 0.23 ± 0.06 | 0.32 ± 0.08 | ns | 9.06 ± 1.57 | 9.84 ± 1.65 |  |
|  | Transport | ** | 1.15 ± 0.12 | 1.39 ± 0.17 | ns | 45.30 ± 2.42 | 43.13 ± 4.06 |  |
|  | Release | ** | 0.29 ± 0.09 | 0.45 ± 0.16 | ns | 11.21 ± 3.00 | 13.40 ± 3.71 |  |
| 4 | Reach | * | 0.49 ± 0.06 | 0.59 ± 0.13 | ns | 24.91 ± 2.60 | 23.45 ± 3.01 |  |
|  | Grasp | ** | 0.15 ± 0.05 | 0.26 ± 0.09 | ns | 7.29 ± 1.71 | 9.89 ± 2.26 |  |
|  | Transport | ** | 1.04 ± 0.12 | 1.23 ± 0.17 | ns | 52.57 ± 2.65 | 49.41 ± 4.14 |  |
|  | Release | ** | 0.31 ± 0.09 | 0.45 ± 0.14 | ns | 15.23 ± 3.74 | 17.26 ± 3.91 |  |
