# Supplementary material for: Gaze and Movement Assessment (GaMA): Inter-site validation of a visuomotor upper limb functional protocol
Source: PLoS One. 2019 Dec 30;14(12):e0219333. doi: 10.1371/journal.pone.0219333 (PMC6936776; doi:10.1371/journal.pone.0219333)
Supplement: S2 Table — For the results of the pairwise comparisons (in column p), * indicates a significant p value less than 0.05, ** indicates a p value less than 0.005, and “ns” indicates a p value that is not significant. (DOCX) [file pone.0219333.s004.docx]

Table S2: Pasta Box Task hand movement values for each movement segment (presented as means ± between-participant standard deviations), with significant results of the pairwise comparisons. For the results of the pairwise comparisons (in column p), * indicates a significant p value less than 0.05, ** indicates a p value less than 0.005, and “ns” indicates a p value that is not significant.

|  | | Hand Distance  Travelled (mm) | | | Hand Trajectory  Variability (mm) | | | Number of  Movement Units | | |
| --- | --- | --- | --- | --- | --- | --- | --- | --- | --- | --- |
| Movement | Movement Segment | *p* | Original | Repeated | *p* | Original | Repeated | *p* | Original | Repeated |
| 1 | Reach-Grasp | ** | 492 ± 26 | 539 ± 36 | * | 19 ± 5 | 30 ± 12 | * | 1.3 ± 0.3 | 1.6 ± 0.4 |
|  | Transport-Release | * | 935 ± 27 | 964 ± 25 | ** | 22 ± 4 | 30 ± 6 | * | 1.2 ± 0.2 | 1.8 ± 0.7 |
| 2 | Reach-Grasp | ns | 505 ± 23 | 506 ± 24 | * | 15 ± 5 | 23 ± 8 | * | 1.0 ± 0.1 | 1.1 ± 0.1 |
|  | Transport-Release | ns | 802 ± 61 | 819 ± 31 | ** | 20 ± 4 | 27 ± 5 | * | 2.3 ± 0.4 | 3.0 ± 0.8 |
| 3 | Reach-Grasp | ** | 746 ± 24 | 796 ± 25 | * | 19 ± 4 | 28 ± 10 | * | 1.1 ± 0.1 | 1.3 ± 0.3 |
|  | Transport-Release | ** | 1186 ± 31 | 1278 ± 52 | ns | 35 ± 8 | 47 ± 18 | * | 1.7 ± 0.4 | 2.4 ± 0.9 |
|  | | Peak Hand  Velocity (mm/s) | | | Percent-to-Peak  Hand Velocity (%) | | |  | | |
| Movement | Movement Segment | *p* | Original | Repeated | *p* | Original | Repeated |  |  |  |
| 1 | Reach-Grasp | ns | 1164 ± 163 | 1098 ± 225 | * | 41.2 ± 4.5 | 45.0 ± 4.0 |  |  |  |
|  | Transport-Release | ns | 1447 ± 136 | 1359 ± 319 | ns | 29.3 ± 3.1 | 27.0 ± 3.6 |  |  |  |
| 2 | Reach-Grasp | ns | 1352 ± 191 | 1200 ± 238 | ns | 36.8 ± 4.4 | 34.3 ± 4.6 |  |  |  |
|  | Transport-Release | ns | 1069 ± 112 | 900 ± 210 | ns | 44.8 ± 8.6 | 44.0 ± 11.8 |  |  |  |
| 3 | Reach-Grasp | ns | 1666 ± 261 | 1585 ± 343 | ns | 35.5 ± 4.0 | 32.4 ± 5.1 |  |  |  |
|  | Transport-Release | ns | 1598 ± 180 | 1477 ± 273 | ns | 36.2 ± 3.8 | 37.8 ± 4.1 |  |  |  |
|  | | Peak Grip  Aperture (mm) | | | Percent-to-Peak  Grip Aperture (%) | | | Percent-to-Peak Hand Deceleration (%) | | |
| Movement | Movement Segment | *p* | Original | Repeated | *p* | Original | Repeated | *p* | Original | Repeated |
| 1 | Reach-Grasp | ns | 116 ± 8 | 109 ± 11 | ns | 73.3 ± 6.5 | 77.8 ± 6.3 | ns | 55.7 ± 8.0 | 58.2 ± 5.4 |
| 2 | Reach-Grasp | ns | 106 ± 10 | 104 ± 11 | ns | 80.1 ± 8.0 | 79.8 ± 8.9 | ns | 72.6 ± 8.6 | 65.1 ± 13.2 |
| 3 | Reach-Grasp | ns | 109 ± 8 | 108 ± 10 | ns | 81.5 ± 4.9 | 83.3 ± 6.7 | ns | 72.8 ± 8.4 | 64.3 ± 13.5 |
