# Supplementary material for: Gaze and Movement Assessment (GaMA): Inter-site validation of a visuomotor upper limb functional protocol
Source: PLoS One. 2019 Dec 30;14(12):e0219333. doi: 10.1371/journal.pone.0219333 (PMC6936776; doi:10.1371/journal.pone.0219333)
Supplement: S3 Table — For the results of the pairwise comparisons (in column p), * indicates a significant p value less than 0.05, ** indicates a p value less than 0.005, and “ns” indicates a p value that is not significant. (DOCX) [file pone.0219333.s005.docx]

Table S3: Cup Transfer Task hand movement values for each movement segment (presented as means ± between-participant standard deviations), with significant results of the pairwise comparisons. For the results of the pairwise comparisons (in column p), * indicates a significant p value less than 0.05, ** indicates a p value less than 0.005, and “ns” indicates a p value that is not significant.

|  | | Hand Distance  Travelled (mm) | | | Hand Trajectory  Variability (mm) | | | Number of  Movement Units | | |
| --- | --- | --- | --- | --- | --- | --- | --- | --- | --- | --- |
| Movement | Movement Segment | *p* | Original | Repeated | *p* | Original | Repeated | *p* | Original | Repeated |
| 1 | Reach-Grasp | ** | 366 ± 52 | 371 ± 34 | * | 16 ± 3 | 21 ± 6 | * | 1.4 ± 0.4 | 1.9 ± 0.5 |
|  | Transport-Release | ns | 646 ± 39 | 652 ± 30 | * | 17 ± 4 | 22 ± 5 | * | 2.3 ± 0.4 | 2.8 ± 0.7 |
| 2 | Reach-Grasp | ** | 456 ± 56 | 516 ± 44 | * | 17 ± 4 | 22 ± 5 | ns | 1.2 ± 0.3 | 1.5 ± 0.4 |
|  | Transport-Release | * | 700 ± 46 | 729 ± 39 | * | 20 ± 5 | 26 ± 6 | * | 2.4 ± 0.4 | 3.1 ± 0.9 |
| 3 | Reach-Grasp | ** | 887 ± 35 | 949 ± 51 | * | 26 ± 5 | 35 ± 11 | ns | 1.6 ± 0.3 | 1.7 ± 0.3 |
|  | Transport-Release | ** | 724 ± 46 | 755 ± 44 | * | 20 ± 4 | 26 ± 6 | * | 2.1 ± 0.6 | 2.7 ± 0.9 |
| 4 | Reach-Grasp | ** | 428 ± 49 | 480 ± 45 | * | 14 ± 4 | 20 ± 7 | * | 1.1 ± 0.2 | 1.4 ± 0.3 |
|  | Transport-Release | ns | 657 ± 46 | 665 ± 34 | * | 20 ± 4 | 27 ± 8 | ns | 2.4 ± 0.4 | 2.8 ± 0.6 |
|  | | Peak Hand  Velocity (mm/s) | | | Percent-to-Peak  Hand Velocity (%) | | |  | | |
| Movement | Movement Segment | *p* | Original | Repeated | *p* | Original | Repeated |  |  |  |
| 1 | Reach-Grasp | ns | 866 ± 166 | 808 ± 191 | ns | 35.2 ± 4.4 | 35.2 ± 5.0 |  |  |  |
|  | Transport-Release | ns | 1042 ± 88 | 931 ± 132 | ns | 21.0 ± 2.6 | 19.9 ± 2.5 |  |  |  |
| 2 | Reach-Grasp | ns | 1149 ± 139 | 1130 ± 267 | * | 30.3 ± 7.2 | 25.0 ± 3.8 |  |  |  |
|  | Transport-Release | ns | 940 ± 70 | 820 ± 107 | ns | 37.7 ± 9.1 | 31.3 ± 8.2 |  |  |  |
| 3 | Reach-Grasp | ns | 1492 ± 187 | 1396 ± 287 | * | 36.3 ± 8.4 | 30.0 ± 4.1 |  |  |  |
|  | Transport-Release | ns | 1009 ± 56 | 883 ± 98 | ns | 24.7 ± 2.4 | 25.0 ± 3.5 |  |  |  |
| 4 | Reach-Grasp | ns | 1157 ± 147 | 1177 ± 230 | ns | 24.5 ± 4.7 | 25.0 ± 5.3 |  |  |  |
|  | Transport-Release | ns | 979 ± 76 | 874 ± 110 | ns | 28.0 ± 7.6 | 30.8 ± 6.7 |  |  |  |
|  | | Peak Grip  Aperture (mm) | | | Percent-to-Peak  Grip Aperture (%) | | | Percent-to-Peak Hand Deceleration (%) | | |
| Movement | Movement Segment | *p* | Original | Repeated | *p* | Original | Repeated | *p* | Original | Repeated |
| 1 | Reach-Grasp | ns | 99 ± 4 | 97 ± 7 | ns | 80.4 ± 4.7 | 77.6 ± 4.2 | ns | 62.0 ± 8.7 | 56.0 ± 9.6 |
| 2 | Reach-Grasp | ns | 114 ± 6 | 114 ± 9 | ns | 73.0 ± 6.3 | 71.4 ± 8.0 | ns | 49.8 ± 6.5 | 46.7 ± 4.6 |
| 3 | Reach-Grasp | ns | 114 ± 7 | 113 ± 7 | ns | 80.4 ± 3.9 | 78.7 ± 5.3 | ns | 61.0 ± 5.3 | 57.4 ± 5.1 |
| 4 | Reach-Grasp | ns | 100 ± 5 | 101 ± 6 | * | 83.7 ± 5.4 | 76.5 ± 7.4 | * | 62.3 ± 13.5 | 50.6 ± 8.7 |
